# Deciphering the code of resistance: a genomic and transcriptomic exploration of the *Cystoisospora suis* Holland-I strain

**DOI:** 10.1038/s41598-025-89372-8

**Published:** 2025-02-14

**Authors:** Teresa Cruz-Bustos, Thomas Eder, Baerbel Ruttkowski, Anja Joachim

**Affiliations:** 1 Institute of Parasitology, Department of Biological Sciences and Pathobiology, University of Veterinary Medicine Vienna, Veterinärplatz 1, Vienna, 1210 Austria; 2 Institute for Medical Biochemistry, Department of Biological Sciences and Pathobiology, University of Veterinary Medicine Vienna, Veterinärplatz 1, 1210 Vienna, Austria

**Keywords:** *Isospora suis*, Coccidia, Swine, Apicomplexa, Toltrazuril, Retrotransposon, Mitochondria, Microbiology, Parasitology, Parasite biology

## Abstract

*Cystoisospora suis*, a member of the apicomplexan order Coccidia and causative agent of neonatal porcine coccidiosis, poses a challenge to pig production due to the emergence of reduced efficacy of toltrazuril, the only EU-approved treatment. To address the critical gaps in understanding toltrazuril resistance and possibilities of early diagnostics, our study investigated the genetic basis of resistance through whole-genome DNA sequencing and transcriptome analysis of two *C. suis* strains, the toltrazuril-susceptible Wien-I and the resistant Holland-I. Additionally, we studied the mitochondrial genome and analysed mitochondrial gene expression in both strains. Our results show that genes encoding proteins involved in host-cell invasion displayed variable expression patterns and genetic mutations, suggesting adaptive changes in invasion mechanisms. Moreover, substantial fluctuations in the expression of genes linked to retrotransposons, accompanied by genetic alterations, were observed, highlighting their potential involvement in genomic rearrangements. Finally, our mitochondrial genome analyses revealed important insights into its genetic organization and conservation. Notably, the marked downregulation of CoI, CoIII and Cytb mRNA levels in the resistant strain Holland-I upon toltrazuril exposure highlights the dynamic response of mitochondrial genes to toltrazuril. These mitochondrial adaptations appear to be closely linked to the parasite drug resistance mechanism, potentially facilitating its survival under pharmacological stress. These findings enhance our knowledge of drug resistance mechanisms in Coccidia and highlight the need for novel management strategies, leading to the development of targeted treatments and controls.

**Supplementary Information:**

The online version contains supplementary material available at 10.1038/s41598-025-89372-8.

## Introduction

*Cystoisospora suis* (syn. *Isospora suis*) is a protozoan parasite of the family Sarcocystidae, order Coccidia, in the phylum Apicomplexa^1^. It infects pigs, particularly suckling piglets, and is a significant cause of neonatal piglet diarrhoea worldwide. The infection can result in non-haemorrhagic diarrhoea and enteritis, leading to adverse health effects such as weight loss and reduced weight gain^2^. The life cycle of *C. suis* is complex and involves both asexual and sexual reproduction stages^1,3^. Infection occurs when piglets ingest oocysts that had previously been shed with the faeces of infected animals into the environment and sporulated to become infectious. Once inside the host’s digestive tract, oocysts release sporozoites which then invade the intestinal epithelial cells. Within the host cells, asexual multiplication occurs, leading to the formation of merozoites. These merozoites continue to differentiate into sexual stages (gamonts and gametes), leading to the formation of oocysts after fusion of micro- and macrogametes^1,4,5^. Porcine cystoisosporosis is commonly controlled by application of the triazinone toltrazuril. However, drug resistance has been reported, and the prevalence of the parasite in pig herds in Europe remains a concern^6,7^. In addition, the increasing pressure to reduce the use of drugs in livestock production highlights the need for alternative control measures and new treatment strategies^8^.

Drug resistance in parasites is a significant and challenging problem in both human and veterinary medicine. Protozoan and metazoan parasites have the ability to adapt and develop resistance to the drugs used to control them. Resistance acquisition mechanisms in apicomplexan parasites pose significant challenges in the treatment of diseases caused by these organisms^9,10^. These parasites have developed various strategies to evade the effects of antiparasitic drugs, leading to treatment failures and public health concerns. One of the primary mechanisms of drug resistance acquisition is through genetic mutations^11^. Mutations can occur in the target gene of antiparasitic drugs, reducing drug binding affinity and rendering it less effective. For example, mutations in the dihydrofolate reductase (DHFR) gene in *Plasmodium* spp. can confer resistance to drugs like pyrimethamine and trimethoprim, which target this enzyme involved in folate metabolism^12,13^. Similarly, mutations in the cytochrome b gene in *T. gondii* and *P. falciparum* can lead to resistance against atovaquone and endochin-like quinolone drugs, a drug that targets the mitochondrial electron transport chain^14–17^. Another common mechanism of drug resistance acquisition is through increased drug efflux. Apicomplexan parasites can upregulate drug transporters, such as ATP-binding cassette (ABC) transporters, which pump the drugs out from the intracellular compartment, thus reducing their effective intracellular concentration. This efflux mechanism has been observed in *Plasmodium* spp., where the overexpression of ABC transporters-like and drug resistance-associated proteins can confer resistance to multiple antimalarial drugs^18^. Furthermore, some apicomplexan parasites can develop resistance by altering their drug targets. For instance, in *Cryptosporidium* spp. the development of resistance was attributed to mutations in the methionyl-tRNA synthetase that led to a change in amino acid sequence, resulting in reduced compound binding while having minimal impact on substrate binding^19^.

The triazine toltrazuril has a historical application as a coccidiocidal drug in veterinary medicine for the control of coccidiosis in chicken, pigs, cattle and dogs^20,21^. Its biochemical action is presumed to block various cellular processes, including the respiratory chain of mitochondria, pyrimidine synthesis, dihydrofolate reductase, and dihydroorotate-cytochrome c reductase^8,22^. Following exposure to toltrazuril, notable enlargement of the perinuclear space, mitochondria, and endoplasmic reticulum was detected in *Eimeria tenella* and *Neospora caninum*^23–25^. Additionally, exposure of *E. tenella* merozoites to the triazine diclazuril^26^, resulted in morphological alterations and diminished the mitochondrial transmembrane potential activity, indicative of the involvement of mitochondria-dependent apoptosis^27^.

Mitochondria are essential subcellular organelles found in almost all eukaryotic cells, primarily responsible for carrying out oxidative metabolism and generating ATP as cellular energy source. Additionally, these organelles play a significant role in the biosynthesis of various cellular components, including pyrimidines, amino acids, phospholipids, nucleotides, folic acid, urea, and diverse metabolites^28^. A remarkable feature of mitochondria is the presence of their own genetic system, complete with the necessary machinery for gene expression, encompassing the synthesis of DNA, RNA, and all the proteins encoded by this second cellular genetic system. Although the mitochondrial genome of Apicomplexa parasites contains a relatively small number of genes, it encodes three proteins vital for mitochondrial respiratory complexes (cytochrome oxidase subunit I, CoI; cytochrome oxidase subunit III, CoIII; and cytochrome b, Cytb), along with fragmented rRNA genes with several rRNA fragments missing^29^. The organization and arrangement of these genes vary across the Apicomplexa, contributing to diverse genome lengths and architectures^30^. Consequently, the precise role of the mitochondrial genome and the intricate mechanisms of gene transcription and translation remain areas of active investigation in Apicomplexa biology^29^.

In this study, we conducted both DNA and RNA sequencing (DNA-seq and RNA-seq) of two strains of *C. suis*, harvested at identical developmental time points in vitro, during asexual multiplication (merogony), to analyse the genetic basis of drug resistance development. This comparative analysis has illuminated significant variations in the genetic repertoires associated with the invasion process and motor activity of the asexual stages, as well as in retrotransposable genetic elements. In conjunction with these techniques, we applied Sanger and Illumina sequencing together with bioinformatic analyses to identify the mitochondrial DNA (mtDNA) sequences of two *C. suis* strains with different toltrazuril susceptibility. This integrative approach led to the complete characterization of the *C. suis* mtDNA, and more notably, pinpointed Cytb and CoI and CoIII as potential molecular targets of toltrazuril. These findings offer critical insights into the mechanisms underlying drug development and environmental adaptation in *C. suis*, highlighting the genetic factors and diversity that may influence its pathogenicity and interaction with host organisms.

## Results

### Genetic variations in annotated genes of *C. suis* merozoites of Holland-I strain

Whole genome DNA sequencing is a robust method for the comprehensive identification of genetic variations such as single and multi-nucleotide polymorphisms (SNPs and MNPs), as well as short insertions and deletions (InDels). To gain more information on the nature of toltrazuril resistance and on the relationship between the two geographically unrelated strains, the susceptible reference strain Wien-I and the resistant isolate, Holland-I, we utilized whole-genome DNA sequencing to identify genetic differences between these two strains. The sequenced reads were aligned against the Wien-I reference strain in Genbank, resulting in a mean genome coverage of 88.72% and 88.48% and a mean depth of 596.36 fold and 688.24 fold for Wien-I and Holland-I, respectively. Through our analysis, we detected a total of 12,917 SNPs, 1,682 MNPs and 640 short InDels, of which 9,771, 1,067 and 513 were found in Holland-I, respectively (See Supplementary Table 1). These annotation of these variants allowed us to link the detected genetic variations to specific genes and potentially elucidate their functional consequences.

**Fig. 1:**
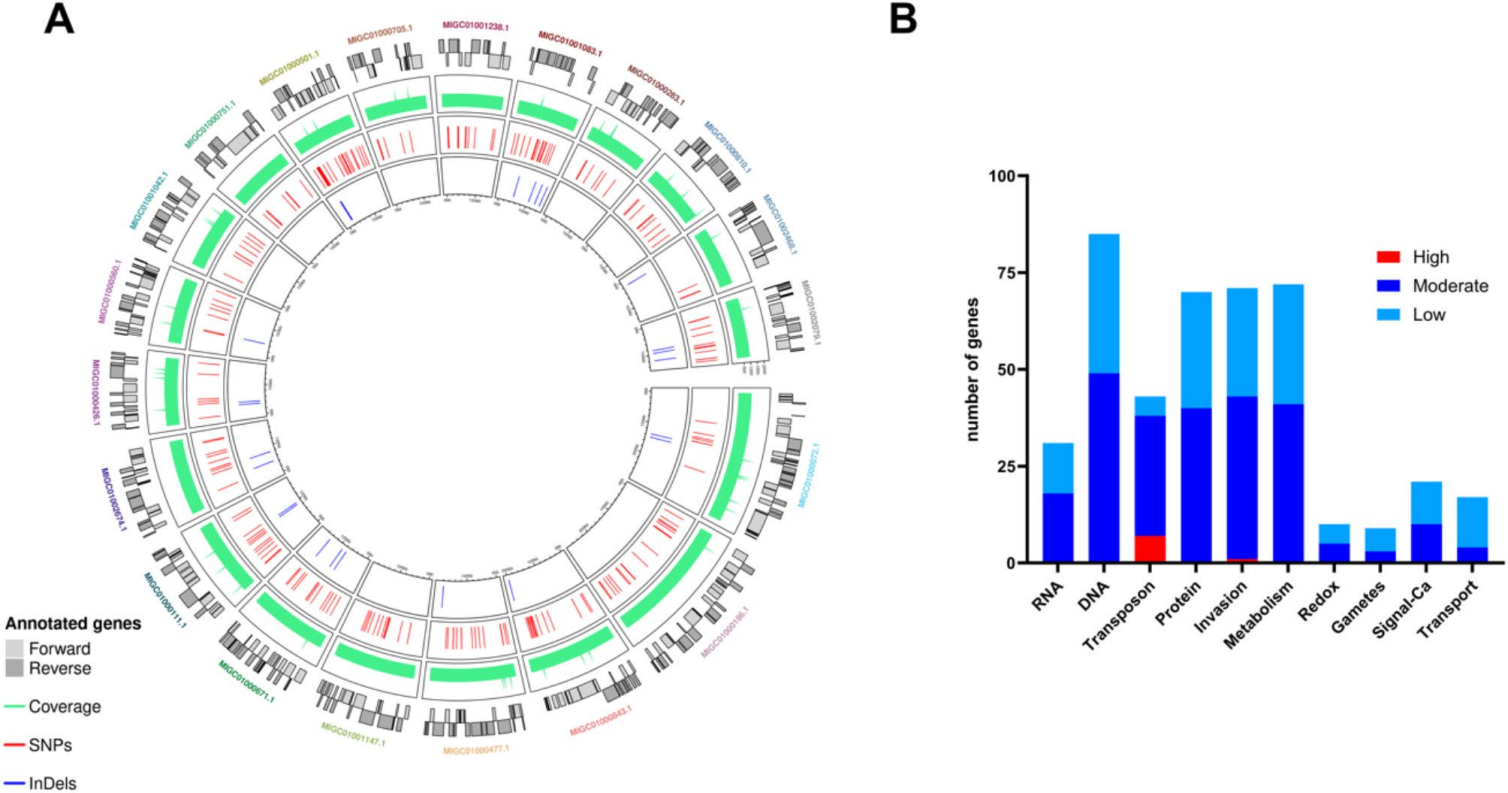
Genetic variation analysis in the toltrazuril-resistant *Cystoisospora suis* Holland-I strain. (A) Comparative circular maps. From the outermost to the innermost track: names of the 20 largest scaffolds of the *C. suis* Wien-I genome assembly; annotated genes shown as grey blocks indicating coding strands; normalized counts per million coverage depicted in green; SNPs and Indels represented by red and blue bars, respectively. (B) Bar chart visualizing the distribution of single nucleotide polymorphisms (SNPs) classified by their variant impact (low, moderate, or high) across different gene functional categories.

We found variations of SNPs and InDels in 4,801 genes in Holland-I. Base on the impact prediction, from these 4,801 genes, 890 were identified to have an impact, 446 with a low (assumed to be mostly harmless or unlikely to change protein behaviour), 560 with a moderate (non-disruptive variant that might change protein effectiveness) and 24 with a high impact (disruptive impact in the protein, probably causing protein truncation, loss of function or triggering nonsense-mediated decay). The genes were categorized based on a synthesis of Gene Ontology (GO) predictions for *C. suis* and orthologs from *T. gondii* as listed in ToxoDB. This categorization was informed by annotations from the KEGG pathway database for *T. gondii*, BLAST homology searches, and insights from recent literature. In our analysis, SNPs were stratified by their variant impacts high, moderate, or low and their distribution across gene categories was documented (Fig. 1, Supplementary Table 2).

The two categories ‘DNA’ and ‘RNA’ contained the highest number of genes with SNPs, predominantly characterized by a low impact on gene function (over 100 genes). This suggests a considerable variation within the DNA/RNA handling machinery that could be of functional significance to the resistance phenotype. In the retrotransposon category, we observed a distinctive SNP distribution pattern: while the minority of genes contained low-impact SNPs (only five of 43 genes), a substantial portion exhibited moderate-impact SNPs, and seven genes were detected with high-impact SNPs. This indicates that genetic variations in transposable elements could be critically disruptive, possibly affecting genomic integrity and contributing to resistance development. However, the moderate-impact SNPs could reflect evolutionary pressure on these elements, which may influence genomic stability and potentially contribute to the development of drug resistance. The ‘Host-cell Invasion’ category revealed a mixture of SNP impacts, including a single gene with a high-impact SNP, while ‘Metabolism’ displayed predominantly low-impact SNPs, except for 42 genes undergoing moderate-impact variations. These findings suggest that genetic alterations in these categories may influence critical biological processes. Categories such as ‘Protein’, ‘Redox’, ‘Gamete’, ‘Signalling’, and ‘Transport’ were characterized by a lower number of SNPs, mostly of low impact. However, the medium number of moderate-impact SNPs in the protein category may reflect selective pressures affecting protein functionality and stability within the resistant strain (Fig. 1b, Supplementary Table 2).

### Illumina sequencing and read mapping of RNA

Transcriptomic analyses were carried out on the asexual merozoite stage of *C. suis* to assess transcript levels. The analysis was conducted on Holland-I strain with and without toltrazuril treatment, and on the Wien-I strain without treatment due to its sensitivity to the drug. The samples yielded an average of 5.3 million sequence reads. Subsequently, the data were aligned to the genome assembly of *C. suis* strain Wien-I. A minimum of 94.58% of the reads per replicate were successfully mapped to the existing *C. suis* genome, facilitating a comprehensive quantitative analysis of gene transcript levels. This robust approach enabled a detailed examination of gene expression levels in the asexual merozoite stage of *C. suis* and provided insights into the molecular mechanisms underlying its biology.

### Identification of differentially expressed genes (DEGs)

**Fig. 2:**
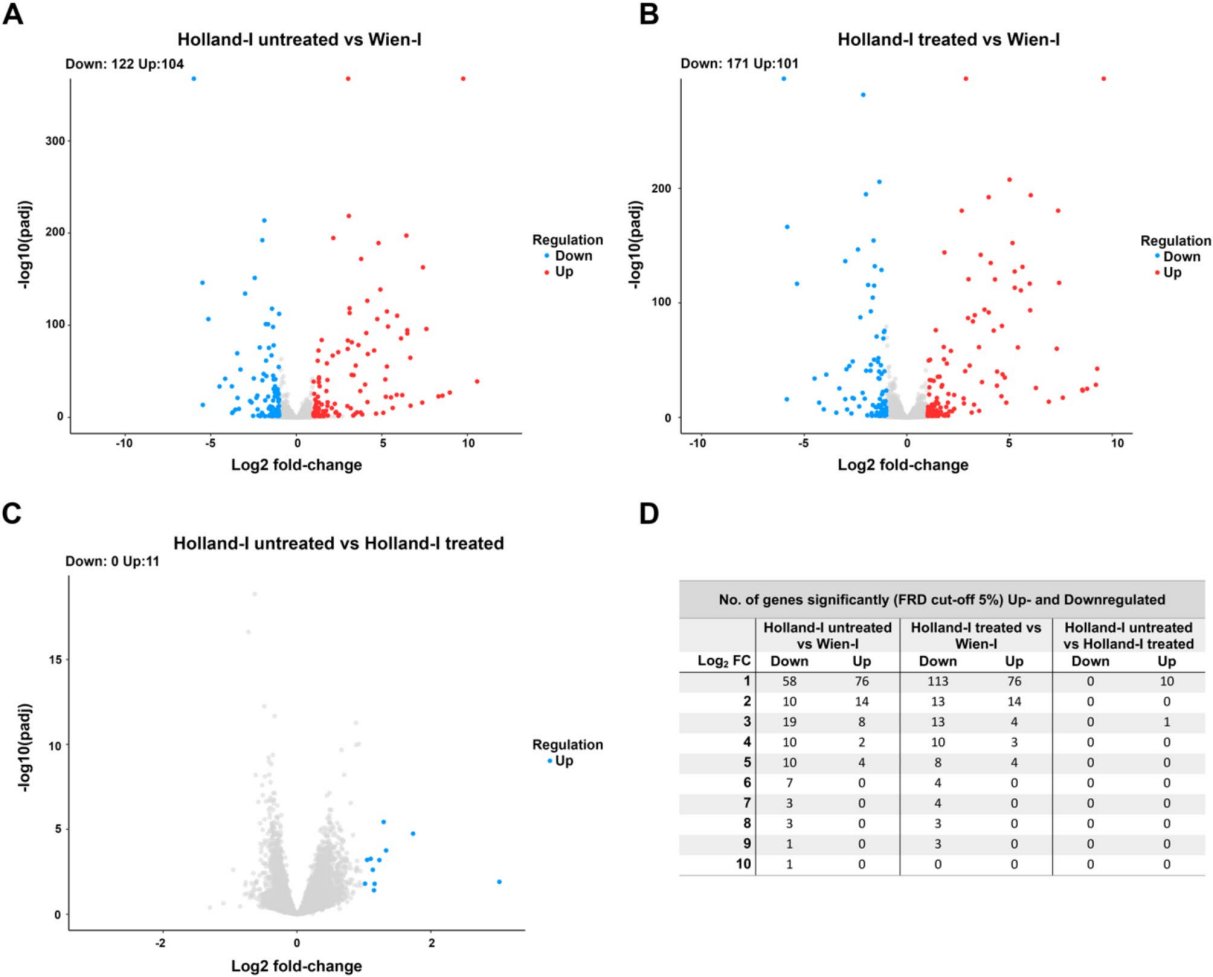
Differential gene expression analysis between Wien-I and Holland-I strains. A to C: Volcano plots of differentially expressed genes in *C. suis* in different strains and under different treatment conditions. Each point represents a single gene; red points indicate significantly upregulated genes, and blue points indicate significantly downregulated genes in Holland-I (toltrazuril-resistant) compared to Wien-I (toltrazuril-susceptible). The x-axis represents the log2 fold change in expression and the y-axis the -log10 of the p-value, indicating the significance of differential expression. (A) Holland-I untreated vs. Wien-I, (B) Holland-I toltrazuril-treated vs. Wien-I, (C) Holland-I untreated vs. Holland-I toltrazuril-treated. D) Summary Table: Number of genes significantly down- and upregulated at various log2 fold change (FC) thresholds for Holland-I untreated or treated vs. Wien-I, as well as Holland-I untreated vs. treated. The padj cutoff is set at 0.05.

The primary objective of this study was to elucidate genes exhibiting altered expression levels in the resistant strain compared to the toltrazuril-susceptible strain of *C. suis*. This comparison aimed to pinpoint genes and proteins potentially associated with drug resistance mechanisms. We employed a threshold for p-adj (adjusted p-value) set at 0.05 and a minimum requirement of an absolute log2 fold change of 1 to identify differentially expressed genes (DEGs). Applying these criteria, a total of 341 DEGs were identified across three distinct comparisons. In the first comparison, analysing the Wien-I versus the Holland-I strain, we identified 122 downregulated and 104 upregulated DEGs in Holland-I. The second comparison, which involved the Wien-I strain and the toltrazuril-treated Holland-I strain, revealed 101 downregulated and 171 upregulated DEGs in Holland-I. The third comparison, between the untreated Holland-I strain and the same strain treated with toltrazuril, yielded 11 downregulated genes in the treated sample, demonstrating significant differences in DEG patterns among the strains and treatment conditions examined. Our analysis identified 101 and 104 upregulated genes in the Holland-I and Holland-I treated samples, when compared to Wien-I (Fig. 2), corresponding to 0.90% and 0.87% respectively of the total predicted *C. suis* genes. Detailed information regarding the identification, characterization, and transcript abundance levels of these genes across the strains, providing insights into the potential genetic underpinnings of toltrazuril resistance, is presented in Supplementary Table 3.

### Dynamics of gene expression

**Fig. 3:**
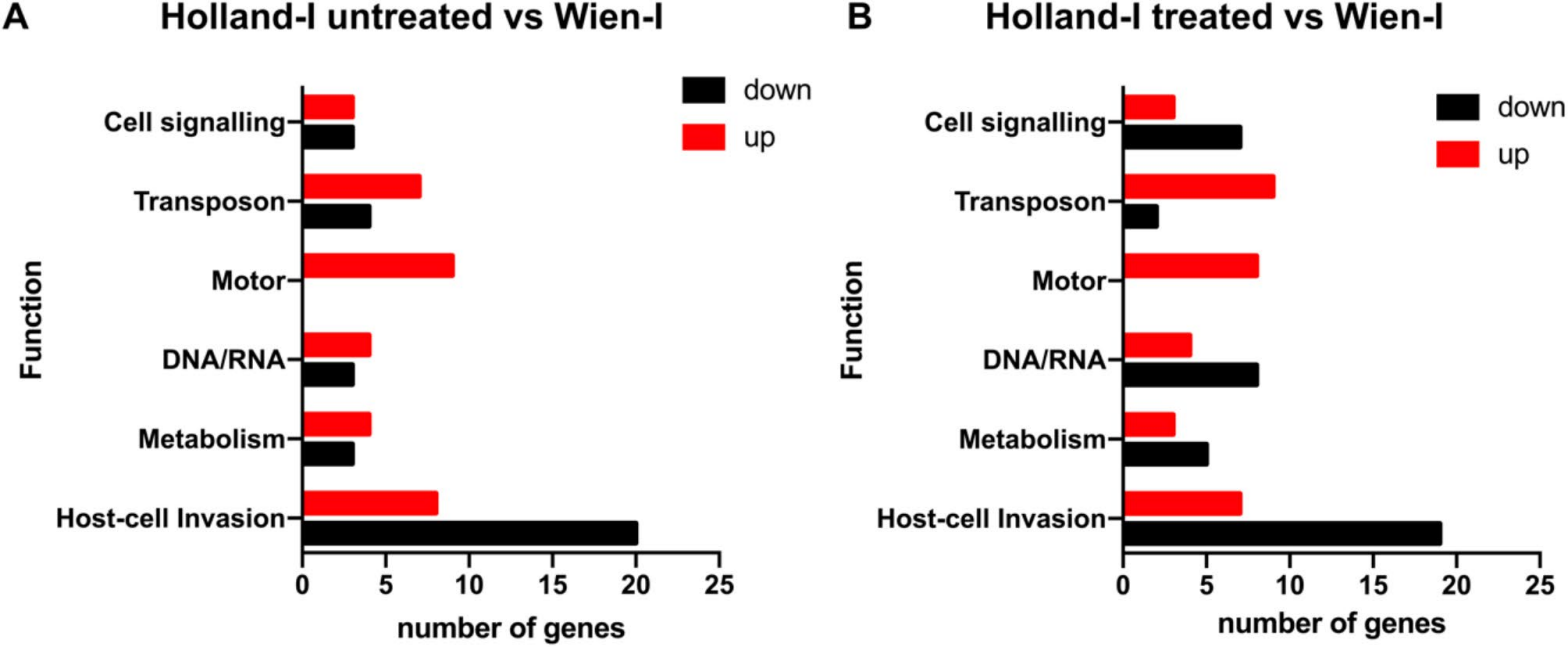
Differential gene expression in *C. suis* strains Holland-I (untreated or toltrazuril-treated) versus Wien-I. The bar chart represents the number of DEGs in the toltrazuril-resistant Holland-I strain relative to the non-resistant Wien-I strain. Each bar indicates the number of DEGs (x-axis) within a specified gene category (y-axis).

A significant proportion of the identified subset of both downregulated and upregulated genes in *C. suis* Holland-I were found to encode proteins of unknown function. From this subset, 11 differentially expressed genes (DEGs) arising from the comparison between the untreated and treated Holland-I groups were excluded from further analysis. Among these excluded DEGs, seven encoded proteins of unknown function, and of the remaining four, only two were unique to this comparison. The genes included in the analysis were categorized based on their functional roles. Notably, genes encoding merozoite proteins, which have been previously characterised and implicated in host cell attachment and invasion, motility, calcium regulation and cell signalling, DNA metabolism and retrotransposon activities, were among the most highly regulated categories (Fig. 3; Table 1).

**Table 1:** List of DEGs identified in this study.

gene_id	Description	Log2FC-Holland-I untreated vs. Wien-I	Log2FC-Holland-I treated vs. Wien-I	log2FC-Holland-I untreated vs. Hol treated	Function
CSUI_007249	formin frm1	n.s.	−1,105909549	n.s.	Actin polimerization
CSUI_005468	sag-related sequence srs60a	−1,013603438	n.s.	n.s.	Adhesion/Invasion
CSUI_009377	sag-related sequence srs44	n.s.	−1,058745132	−1,160019164	Adhesion/Invasion
CSUI_004246	sag-related sequence srs26j	−1,328994698	−1,114820384	n.s.	Adhesion/Invasion
CSUI_005667	sag-related sequence srs28	−1,109645493	−1,507632787	n.s.	Adhesion/Invasion
CSUI_003350	srs domain-containing protein	1,105690256	1,408228675	n.s.	Adhesion/Invasion
CSUI_005472	sag-related sequence srs60a	1,782494486	1,887198443	n.s.	Adhesion/Invasion
CSUI_007678	sag-related sequence srs53f	−7,604787526	−7,415420127	n.s.	Adhesion/Invasion
CSUI_007676	sag-related sequence srs53c	−7,405525206	−7,367358694	n.s.	Adhesion/Invasion
CSUI_004444	sag-related sequence srs26i	−5,894887765	−5,631574395	n.s.	Adhesion/Invasion
CSUI_003351	srs domain-containing protein	−5,299945055	−5,25094821	n.s.	Adhesion/Invasion
CSUI_007679	sag-related sequence srs53a	−4,917517246	−5,147397997	n.s.	Adhesion/Invasion
CSUI_011314	sag-related sequence srs53f	−3,47414931	−3,513240899	n.s.	Adhesion/Invasion
CSUI_010484	sag-related sequence srs53f	−3,119742463	−3,595547427	n.s.	Adhesion/Invasion
CSUI_009012	srs domain-containing protein	−3,072558598	−2,672286866	n.s.	Adhesion/Invasion
CSUI_007477	sag-related sequence srs53c	−1,821837574	−1,945623076	n.s.	Adhesion/Invasion
CSUI_005474	sag-related sequence srs60a	−1,367113535	−1,30783549	n.s.	Adhesion/Invasion
CSUI_003091	sag-related sequence srs17b	−1,122125404	n.s.	n.s.	Adhesion/Invasion
CSUI_011375	srs domain-containing protein	−1,087311522	n.s.	n.s.	Adhesion/Invasion
CSUI_008107	sag-related sequence srs17a	1,599059396	1,755737692	n.s.	Adhesion/Invasion
CSUI_003818	srs domain-containing protein	1,867038531	2,123748282	n.s.	Adhesion/Invasion
CSUI_009667	egf family domain-containing protein	n.s.	1,093572566	n.s.	Cell signaling
CSUI_010999	camp-dependent protein kinase regulatory	n.s.	−1,226812411	n.s.	Cell signaling
CSUI_000404	Calcium signaling protein kinase mark	−1,12276393	−1,04193649	n.s.	Cell signaling
CSUI_001240	ef hand domain-containing protein	n.s.	−1,335008735	n.s.	Cell signaling
CSUI_002354	ef hand family protein	n.s.	−1,334110637	n.s.	Cell signaling
CSUI_005651	ef hand domain-containing protein	n.s.	−1,427846448	n.s.	Cell signaling
CSUI_002874	Calcium-dependent protein kinase cdpk4a	−1,292592935	−1,31873742	n.s.	Cell signaling
CSUI_008474	Calcium-dependent protein kinase cdpk4a	−1,270724328	−1,288347597	n.s.	Cell signaling
CSUI_009777	Polycystin cation channel protein	1,080856263	1,279074593	n.s.	Cell signaling
CSUI_010565	Calcium binding egf domain-containing protein	1,586986816	n.s.	n.s.	Cell signaling
CSUI_008347	Calcium binding egf domain-containing protein	1,813471192	1,117047366	n.s.	Cell signaling
CSUI_007090	Pan domain-containing protein	−3,633128797	−3,986119921	n.s.	Host cell-attachment/Invasion
CSUI_004141	Pan domain-containing protein	−3,112879465	−3,003152896	n.s.	Host cell-attachment/Invasion
CSUI_006321	Microneme protein mic4	1,024277794	1,145717201	n.s.	Host cell-attachment/Invasion
CSUI_006151	Microneme protein 13	1,445113931	1,078766881	n.s.	Host cell-attachment/Invasion
CSUI_006388	Apical membrane antigen 1 protein	n.s.	−1,232632589	−1,734768611	IMC
CSUI_002065	Serine threonine-protein (rop37)	−4,182033123	−4,230242076	n.s.	Invasion/Virulence
CSUI_002064	Rhoptry kinase family protein rop37 (incomplete catalytic triad)	−4,02326811	−4,394757545	n.s.	Invasion/Virulence
CSUI_005019	Rhoptry kinase family protein rop37 (incomplete catalytic triad)	−1,12800539	−1,094366929	n.s.	Invasion/Virulence
CSUI_011499	Thrombospondin type 1 domain-containing protein	1,418486426	n.s.	n.s.	Invasion/Virulence
CSUI_001983	Thrombospondin type 1 domain-containing	1,764683932	1,597651892	n.s.	Invasion/Virulence
CSUI_006910	Flagellar associated protein	−1,016661539	n.s.	n.s.	Microgametes
CSUI_011137	Dynein gamma flagellar outer	n.s.	1,018653589	n.s.	Microtubule motor activity
CSUI_004829	Dynein gamma flagellar outer	1,056262516	n.s.	n.s.	Microtubule motor activity
CSUI_007938	Dynein gamma flagellar outer	1,462432172	1,089982645	n.s.	Microtubule motor activity
CSUI_005475	Dynein gamma flagellar outer	2,105109763	n.s.	n.s.	Microtubule motor activity
CSUI_008375	Dynein heavy chain family protein	3,26019502	2,942806161	n.s.	Microtubules
CSUI_009281	Dynein heavy chain family protein	2,274024378	2,155057535	n.s.	Microtubules
CSUI_010528	Dynein heavy chain family protein	2,330538627	2,051372122	n.s.	Microtubules
CSUI_009772	Dynein heavy chain related	2,61204312	2,645999094	n.s.	Microtubules
CSUI_004124	Dynein heavy chain family protein	2,699763506	2,582549465	n.s.	Microtubules
CSUI_003185	Dynein heavy chain family protein	3,763776521	3,285450009	n.s.	Microtubules
CSUI_009198	Retrotransposon gag protein	−6,486405962	−6,00608402	n.s.	Retrotransposon
CSUI_006462	Retrotransposon ty3-gypsy subclass	5,461776131	5,845415341	n.s.	Retrotransposon
CSUI_009884	Retrotransposon ty3-gypsy subclass	n.s.	1,235948754	n.s.	Retrotransposon
CSUI_010377	Retrotransposon ty3-gypsy subclass	n.s.	1,319513604	n.s.	Retrotransposon
CSUI_002784	Retrotransposon ty3-gypsy subclass	1,558644484	1,312017113	n.s.	Retrotransposon
CSUI_010574	Retrotransposon ty3-gypsy subclass	2,059366979	n.s.	n.s.	Retrotransposon
CSUI_005100	Retrotransposon ty3-gypsy subclass	5,137431242	5,34999588	n.s.	Retrotransposon
CSUI_006463	Retrotransposon ty3-gypsy subclass	5,479810194	5,825029257	n.s.	Retrotransposon
CSUI_001909	dna rna polymerases superfamily protein	−6,435304941	n.s.	n.s.	Retrotransposon
CSUI_011456	Retrotransposon nucleocapsid related	n.s.	1,335066328	n.s.	Retrotransposon
CSUI_001910	Hypothetical protein	−6,664187101	−7,300251604	n.s.	Retrotransposon
CSUI_000007	Retrotransposon ty3-gypsy subclass	1,000693387	1,000693387	n.s.	Retrotransposon
CSUI_009428	gag-pol fusion protein	−1,12578305	n.s.	n.s.	Retrotransposons
CSUI_005489	gag-pol fusion protein	1,232676389	1,266042242	n.s.	Retrotransposons

The genes are listed along with their annotation number in ToxoDB, gene name, biological function, and gene abundance (LogFC), in each comparison. Negative values indicate a downregulation.

**Fig. 4:**
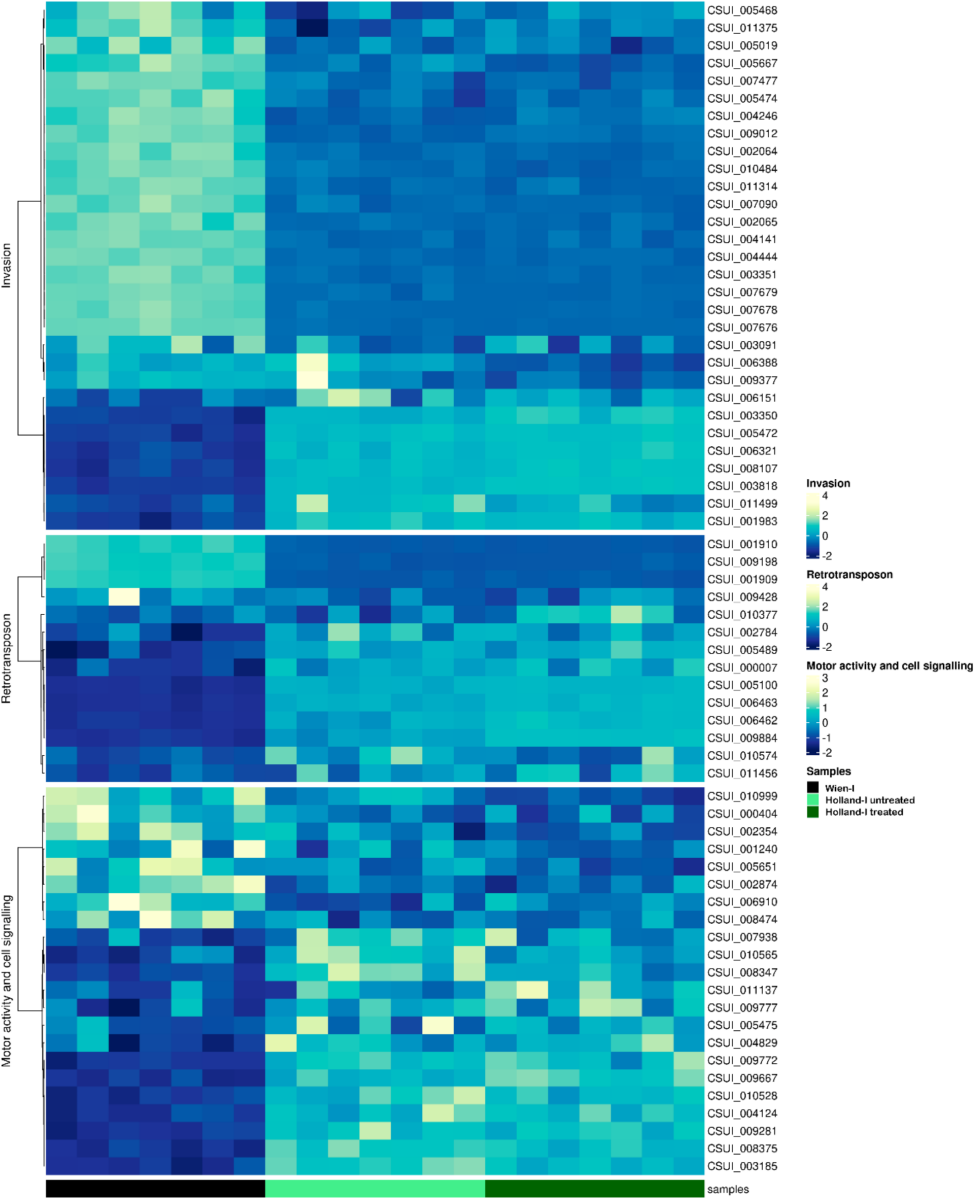
Heatmap of row-wise z-transformed gene expression across three groups: Wien-I, Holland-I untreated and Holland-I toltrazuril-treated, with unsupervised clustering. The top panel shows DEGs associated with the host invasion process; the bottom panel shows DEGs associated with motor activity and cell signalling; and the middle panel shows retrotransposon-related DEGs. Each row represents a gene, with expression levels indicated as follows: higher than average in yellow, average in light blue and lower than average in blue. Each column represents a sample, representing the seven biological replicates of Wien-I, Holland-I untreated and Holland-I toltrazuril-treated.

Within the Host-cell Invasion category, the Holland-I strain exhibited 20 downregulated and eight upregulated DEGs in comparison to the susceptible Wien-I strain. Furthermore, when the Holland-I strain was treated with toltrazuril, 19 DEGs were downregulated and seven upregulated relative to Wien-I. In the Holland-I strain, upregulation of two genes encoding distinct microneme proteins (MICs), alongside an increased expression of two thrombospondin type 1 domain-containing proteins was observed. In contrast, two genes associated with PAN-domain proteins and three rhoptry proteins (ROPs) were found to be downregulated. Additionally, we identified 20 surface antigen (SAG) and SAG-related sequence (SRS) proteins, four of which showing upregulation, while eight exhibited significant downregulation, featuring a log fold change ranging from 3 to 7. We observed that, within the Motor category, 10 DEGs corresponding to dynein family proteins exhibited upregulation in Holland-I. Additionally, in the Cell Signalling category, we identified 11 DEGs, out of which only three showed increased expression while the remaining eight were downregulated. Our analysis also revealed six DEGs within the RNA category and eleven DEGs in the DNA category. Notably, within these identified genes, four are commonly associated with chromatin-associated proteins. We also identified 14 genes associated with transposable elements with high levels of differential regulation (Fig. 4). In ToxoDB, filtering by InterPro domains, a total of 155 genes were identified as retrotransposon-related genes. Among them, 102 belong to the Eimeriidae family (101 in the genus *Eimeria* and one in the genus *Cyclospora*), and 53 to the Sarcocystidae family (all from 53 *C. suis*; no hits for the genera *Toxoplasma*,* Hammondia*,* Neospora*,* or Sarcocystis*).

### Correlation between DEGs and SNPs

To investigate the relationship between transcriptomic alterations and SNP variations within the Holland-I and Wien-I strains, we conducted a comparative analysis focusing on DEGs that exhibited SNPs of varying impact levels. This comparative approach underscores the potential for DNA-level genetic variations to drive changes in gene expression, thereby influencing phenotypic outcomes. We identified 24 DEGs with such variations: one gene with high-impact SNPs, five genes with low-impact SNPs, and 19 genes with moderate-impact SNPs. Notably, these genes were predominantly associated with processes related to cellular invasion and retrotransposon activity (Table 2).

**Table 2:** List of differentially expressed genes (DEGs) with single nucleotide polymorphisms (SNPs) identified in this study.

gene_id	Description	Log2FC-Holland-I untreated vs. Wien-I	Log2FC-Holland-I treated vs. Wien-I	Log2FC-Holland-I untreated vs. Hol treated	Function	Variation impact
CSUI_004246	sag-related sequence srs26j	−1,328994698	−1,114820384	n.s.	Adhesion/Invasion	Moderate
CSUI_005667	sag-related sequence srs28	−1,109645493	−1,507632787	n.s.	Adhesion/Invasion	Moderate
CSUI_009377	sag-related sequence srs44	n.s.	−1,058745132	−1,160019164	Adhesion/Invasion	Moderate
CSUI_005468	sag-related sequence srs60a	−1,013603438	n.s.	n.s.	Adhesion/Invasion	Moderare
CSUI_005472	sag-related sequence srs60a	1,782494486	1,887198443	n.s.	Adhesion/Invasion	Moderate
CSUI_003350	srs domain-containing protein	1,105690256	1,408228675	n.s.	Adhesion/Invasion	Moderate
CSUI_000404	Calcium signaling protein kinase mark	−1,12276393	−1,04193649	n.s.	Cell signaling	Moderate
CSUI_010999	Camp-dependent protein kinase regulatory	n.s.	−1,226812411	n.s.	Cell signaling	Low
CSUI_009667	egf family domain-containing protein	n.s.	1,093572566	n.s.	Cell signaling	Low
CSUI_006910	Flagellar associated protein	−1,016661539	n.s.	n.s.	Microgametes	Moderate
CSUI_008375	Dynein heavy chain family protein	3,26019502	2,942806161	n.s.	Microtubules	Low
CSUI_006230	Alaserpin isoform x2	1,419526009	1,621707239	n.s.	Non identified function	Moderate
CSUI_006911	wd g-beta repeat-containing protein	−1,127412107	n.s.	n.s.	Protein binding	Moderate
CSUI_003321	Iron-containing superoxide dismutase	−3,237290598	−3,061790773	n.s.	REDOX	Low
CSUI_001909	dna rna polymerases superfamily protein	−6,435304941	n.s.	n.s.	Retrotransposon	Moderate
CSUI_009198	Retrotransposon gag protein	−6,486405962	−6,00608402	n.s.	Retrotransposon	High
CSUI_006462	Retrotransposon ty3-gypsy subclass	5,461776131	5,845415341	n.s.	Retrotransposon	Low
CSUI_009884	Retrotransposon ty3-gypsy subclass	n.s.	1,235948754	n.s.	Retrotransposon	Moderate
CSUI_010377	retrotransposon ty3-gypsy subclass	n.s.	1,319513604	n.s.	Retrotransposon	Moderate
CSUI_002784	Retrotransposon ty3-gypsy subclass	1,558644484	1,312017113	n.s.	Retrotransposon	Moderate
CSUI_010574	Retrotransposon ty3-gypsy subclass	2,059366979	n.s.	n.s.	Retrotransposon	Moderate
CSUI_005100	Retrotransposon ty3-gypsy subclass	5,137431242	5,34999588	n.s.	Retrotransposon	Moderate
CSUI_006463	Retrotransposon ty3-gypsy subclass	5,479810194	5,825029257	n.s.	Retrotransposon	Moderate
CSUI_009428	gag-pol fusion protein	−1,12578305	n.s.	n.s.	Retrotransposons	Moderate

Table legend: this table lists DEGs identified in this study, along with the following details for each gene: annotation number in ToxoDB, gene name, biological function, variation impact, and gene abundance (LogFC) in each comparison. Negative LogFC values indicate downregulation.

### Mitochondrial genome organization

**Fig. 5:**
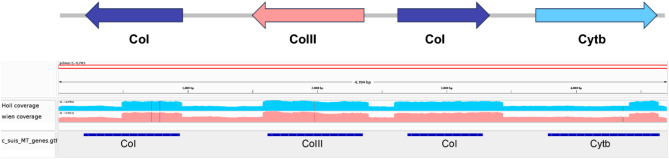
PCR amplification results and subsequent comparative Illumina DNA sequencing of mitochondrial genes from two *C. suis* strains. The top panel displays a schematic representation of the mitochondrial genome with arrows indicating the direction of transcription. The middle panel presents the PCR coverage, with the solid red line indicating normalized read coverage, ensuring the detection of homologous sequences between strains. The blue shaded regions represent the extent of PCR amplification for each gene. The lower panel provides a comparative view coverage; regions of high sequence identity with the reference genome are marked in lighter blue for Holland-I and pink for Wien-I.

The mtDNA genome information published for other members of the Coccidia, several *Eimeria* spp. and *T. gondii*, served as a template for designing PCR primers that carefully avoided nuclear genome sequences. Primer pairs often generated single amplicons by PCR that, when sequenced and annotated, revealed a high level of sequence identity to each other at the beginning or the end of the read regions, suggesting that the mitochondrial genomes may be either linearly concatenated or circular in nature, enabling successful PCR amplification of nearly full-length mt genomes. Despite the puzzling nature of the PCR results, all the contigs generated by sequencing successfully assembled into a single mtDNA sequence and the two complete mitochondrial genomes sequences of both strains obtained through direct sequencing of PCR products displayed identical lengths. The mitochondrial genome of *C. suis* spans 4,703 bp and is circularly mapped, although its physical form is not yet fully determined (Supplementary Data 1). The *C. suis* mt genome exhibits a specific organization, containing portions of CoI, or in some cases, complete cytochrome genes (CoIII and Cytb) interspersed with five fragments of large subunit rDNA and four fragments of small subunit rDNA (Fig. 5). Notably, the mitochondrial genome displays a strong bias towards A and T nucleotides, with A accounting for 30% (1432 bp) and T for 34% (1524 bp), while G and C represent 18% each (877 and 870 bp, respectively).

To facilitate whole genome alignments, we linearized all mitochondrial genome sequences at the same position. Interestingly, no intraspecific variation was observed between the two strains, as they shared 99.87% identity. To further our analysis, we performed an alignment of the DNA sequencing reads against the mitochondrial genome of 4,703 base pairs in length. This comparison revealed the presence of five single nucleotide polymorphisms (SNPs) when compared to the reference strain Wien-I; interestingly, these SNPs were consistent across both strains studied. In addition, the sequencing coverage for both strains was remarkably similar, as shown in Fig. 5. This finding suggests a high level of conservation among the strains, indicating a stable and well-maintained mitochondrial genome. All genes within the mitochondrial genomes were found to have stop codons. Moreover, each sequence commenced with a methionine and concluded with a canonical stop codon.

### Quantitative RT-PCR

**Fig. 6:**
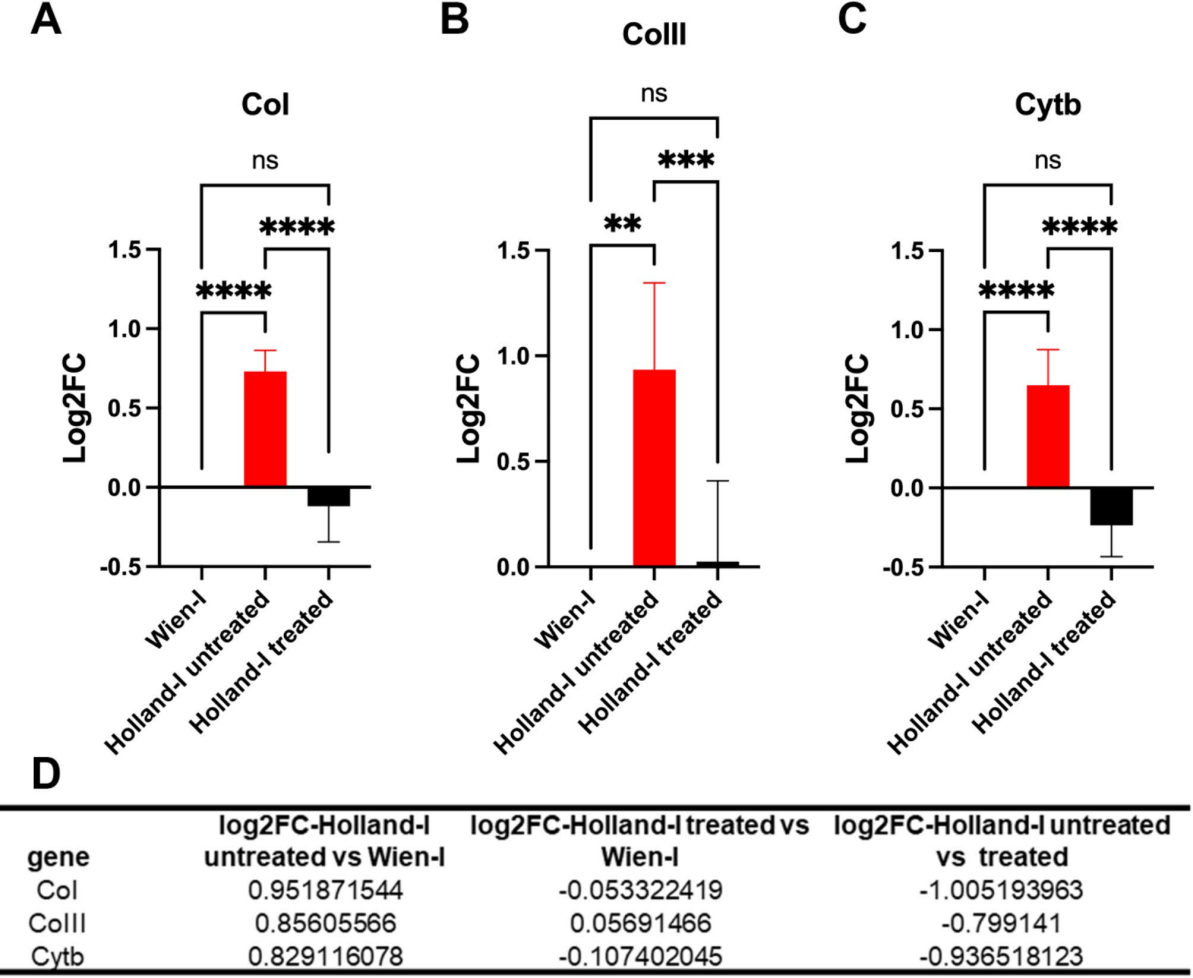
Relative mRNA levels of mitochondrial genes CoI (A), CoIII (B) and Cytb (C). qRT-PCR shows upregulation of all three genes in Holland-I compared to the toltrazuril-susceptible Wien-I and down-regulation in Holland-I under treatment. Values represent the mean ± standard error (SE) (*n* = 3). Glyceraldehyde-3-phosphate and actin were used for normalization. One-way ANOVA with multiple comparisons. Asterisks represent significant difference *P < 0.05, **P < 0.01***, *P* < 0.001, ****P < 0.0001. D) Summary of the differential gene expression analysis according to RNA-seq analysis.

Quantitative RT-PCR analysis was conducted to assess the RNA levels of *C. suis* CoI and CoIII and Cytb. The transcripts levels were calculated according to the 2-ΔΔCt values using glyceraldehyde-3-phosphate (GAPDH) and actin as a reference genes. Our data demonstrated a significant upregulation of CoI, CoIII and Cytb mRNA in untreated Holland-I relative to Wien-I, indicating differential expression associated with the resistance phenotype. In contrast, when evaluating the impact of drug treatment on the mRNA expression of Holland-I, we observed a noticeable downregulation in the levels of CoI, CoIII and Cytb after a 24-hour treatment period compared to the untreated Holland-I. This decrease in expression post-treatment suggests a responsiveness of these genes to treatment, further supporting their potential mechanistic role in the resistance of Holland-I to toltrazuril. The expression levels determined by qRT-PCR were consistent with those obtained by RNA-seq (Fig. 6), confirming the accuracy and reliability of the results.

## Discussion

The emergence and spread of drug-resistant strains of coccidian parasites poses a major challenge to current therapeutic strategies and calls for elucidating the underlying genetic mechanisms of resistance. Especially in the case of *C. suis*, where toltrazuril is the only registered effective drug and has now been used for decades to control suckling piglet coccidiosis, such information could provide insights on how to determine the presence of resistant isolates in the field and how to overcome poor treatment efficacy due to toltrazuril resistance. To address this, our study involved genome and transcriptome analyses of two unrelated *C. suis* isolates: the toltrazuril-resistant Holland-I^6^ and the toltrazuril-susceptible Wien-I^31^. Utilizing whole-genome DNA sequencing and Sanger sequencing of the mitochondrial genome, we identified genetic variations that provide insights into the organization and potential adaptive mechanisms of the toltrazuril-resistant *C. suis* strain Holland-I. Differential gene expression was evaluated through RNA-seq and RT-qPCR analyses of both strains, as well as the Holland-I strain with and without toltrazuril treatment. The latter analysis could not be conducted for Wien-I due to its sensitivity to the drug^32^. A critical consideration in our study was the need to replicate in vivo treatment conditions to ensure biologically relevant findings. Parasites were treated with toltrazuril over an extended period to allow sufficient time for egress and collection of intact parasites, which are essential for high-quality RNA and DNA sequencing. Short-term drug exposure is not only insufficient to mimic in vivo pharmacodynamics but also results in rapid disintegration of highly susceptible strains, such as Wien-I, making it technically challenging to obtain reliable gene expression data. This is consistent with findings from similar studies^23,24^, where prolonged drug incubation times were necessary to capture meaningful effects on parasites at critical developmental stages. For example, *Plasmodium falciparum* cultures were incubated for 48 h at the trophozoite stage^33^, and *Eimeria tenella* parasites were exposed to drugs over successive cycles to study resistance^34,35^ and *Toxoplasma gondii* tachyzoites were treated until egress to ensure intact material for molecular analyses^36^. Such approaches underscore the importance of extended exposure to fully observe drug effects and ensure representative sampling for downstream analyses.

While our approach provides a perspective on the genetic basis of toltrazuril resistance, we recognize the limitation of using only two isolates, as observed differences may reflect isolate-specific variation rather than definitive markers of resistance. In order to generalise the present findings future research will include geographically different isolates collected in the field and tested in vitro and in vivo for resistance of susceptibility to toltrazuril as described for the two strains used in the present study to be able to define common genetic traits which could further serve in the future as possible markers applicable in the field. We have chosen not to focus our discussion on the DEG comparison between treated and untreated parasites due to limitations in the functional annotation of these genes. In particular, 7 of the 11 differentially expressed genes in this comparison are uncharacterised, making it difficult to draw meaningful conclusions about their potential role in toltrazuril resistance. Although the remaining four genes have known functions, this small subset alone does not provide a robust basis for hypothesising resistance mechanisms. Including this comparison would risk speculative interpretations that could detract from the clarity of our overall findings.

Overall, the results of this study highlight several aspects of the genetic and molecular mechanisms underlying drug resistance in *C. suis*. Our comprehensive genomic and transcriptomic analyses revealed distinct patterns of genetic variation and differential gene expression between the two strains that could be related to toltrazuril susceptibility or resistance, underscoring the adaptive strategies employed by the parasite to reduce or evade drug efficacy. Correlation of transcriptomic data with SNP analysis in the Holland-I strain provided insight into the functional consequences of genetic variation. In addition, detailed analysis of the mitochondrial genome and its gene expressions adds another dimension to the complexity of the resistance phenotype.

1. Host cell invasion, motor activity and cell signalling.

Apicomplexan parasites secrete a diverse array of proteins to facilitate host cell invasion and to modulate host protein expression^37–43^. The transcriptional downregulation in key protein families such as PAN/Apple domain proteins, and ROPs in the Holland-I strain could signify a strategic adaptation to circumvent host immune defences or an indication of evolving drug resistance mechanisms. The SAGs and SRS proteins, known for their role in host cell adhesion and immune modulation^42^, showed downregulation and genetic variations which could imply a stealth strategy adopted by the Holland-I strain, potentially aiding in its survival and persistence in the face of host defences and pharmacological interventions. Their downregulation in the Holland-I strain suggests a reduced interaction of merozoites from this strain with host cells and the host’s immune system, as compared to the Wien-I strain.

Significantly, the variations in genes associated with host-cell invasion not only suggest mechanisms of adaptive evolution in the response to drug exposure, but also highlight potential targets for novel therapeutic interventions. The observed genetic mutations and the diverse expression patterns in these genes indicate a strategic modification of invasion pathways, which could be pivotal in developing resistance. This adaptation may facilitate the survival by altering its interaction with host cellular mechanisms, a finding that aligns with previous studies on coccidian survival strategies under drug pressure^34^.

Dynein proteins and genes associated with calcium-dependent proteins serve distinct yet potentially intersecting roles within cellular processes. Dynein proteins are motor proteins that facilitate movement along microtubules through ATP hydrolysis^44^, playing crucial roles in vesicular transport^45^, organelle positioning, spindle assembly, and chromosome segregation during cell division^46^. On the other hand, calcium-dependent protein kinases (CDPKs) are activated by calcium ions and are instrumental in various cellular functions, including signal transduction pathways that regulate cell motility, host cell invasion, and cell cycle progression^47–49^. The upregulation of dynein proteins alongside the downregulation of certain CDPKs and calcium-binding proteins in the resistant Holland-I strain suggests a complex adaptive mechanism. The increased expression of dynein proteins could represent a compensatory mechanism aimed at enhancing cellular transport and trafficking, crucial for the parasite survival under drug pressure. This might involve mechanisms for drug sequestration or expulsion to circumvent drug effects. Conversely, the reduction in CDPKs and calcium-binding proteins might reflect an adjustment in calcium-dependent signalling pathways, critical for motility, invasion, and cell cycle control, indicating an evolutionary adaptation to the stress induced by drug exposure. Interestingly, bumped-kinase inhibitor 1369, an anticoccidial compound which binds to *C. suis* CDPK1 is able to inhibit the development of both Wien-I and Holland I^50^, indicating that the involvement of CDPKs in toltrazuril resistance still needs to be investigated in more detail.

2. Retrotransposon elements.

The regulatory patterns and genetic variations observed in genes related to transposable elements suggest a significant role for retrotransposons. Retrotransposons, or transposable elements, are mobile genetic elements that shape genome structure and evolution. Their movement and insertion into different genomic positions can have notable functional impacts^51,52^. Retrotransposon activity is influenced by environmental factors, such as drug exposure, which can alter their activity and impact the genetic landscape, potentially contributing to drug resistance^53^. Retrotransposons can influence the development and spread of resistance through several mechanisms^54^. One primary mode is their insertion near or within genes crucial for drug metabolism or as drug targets. These insertions can alter gene function, leading to changes in drug metabolism or sensitivity, resulting in drug resistance. Retrotransposon activity can also facilitate genetic exchange, contributing to the spread of resistance within a population^55,56^.

Another factor contributing to drug resistance in parasites is their rapid reproduction, which generates genetic diversity^57^, allowing drug-resistant mutants to emerge and become predominant. Apicomplexan parasites undergo sexual reproduction, leading to genetic recombination and further diversification^58,59^, which may result in novel functions impacting drug resistance-related genes^13^. This makes the analysis of the associations between retrotransposon insertions and genes particularly intriguing, as evidenced in the Holland-I strain of *C. suis*.

Although active Ty3/Gypsy retrotransposons are not observed in all Coccidia investigated so far, multiple sequences derived from Ty3/Gypsy exist^60^. A possible explanation for the elevated presence and activity of retrotransposons in *C. suis*, compared to other members of the Sarcocystidae, could be attributed to its life cycle. In a single host, *C. suis* undergoes few mitotic generations followed by meiosis, while e.g. *T. gondii* undergoes mitosis frequently across intermediate hosts and meiosis only in the definitive host^60,61^. During meiosis, the genome is subject to extensive recombination and rearrangement, which is essential for offspring diversity. Retrotransposons can influence this process by inserting into the genome and causing mutations, altering gene expression, and leading to genetic variation in gametes^62^. Additionally, they can act as sources of new genetic material, incorporated into functional genes or regulatory regions during meiosis through a process known as exon shuffling^63^. This can result in the creation of novel genes with new functions, contributing to the evolution of new traits and biological processes^62,64^. Furthermore, the significant fluctuations in the expression of genes associated with retrotransposons, accompanied by notable genetic variation, point to their role in genomic rearrangements in Holland-I. These rearrangements could be critical for the development of drug resistance.

3. Mitochondrion.

Mitochondrial genome content and structure vary widely across the Apicomplexa. While the mtDNAs of all sequenced apicomplexans share the characteristic of having just three protein-coding genes (CoI, CoIII, and Cytb) and exhibit rRNA gene fragmentation, there is considerable diversity within the phylum in terms of gene orientation, arrangement, and overall genome structure^30,65^. Our mitochondrial genome analysis revealed a genome that maps circularly and encompasses 4,703 bp, smaller than those of other Apicomplexa with an average of 6 kbp. The genome-specific arrangement produced contigs containing two portions of CoI with different transcriptional directions. Additionally, the contigs included the complete cytochrome genes CoIII and CytB, with CoIII inserted between the two CoI fragments and CytB following the second CoI fragment, the two of them sharing the same transcriptional direction. Additionally, these contigs were interspersed with mtDNA rRNA gene fragments. The number, direction and topology of the three genes were almost identical in the two mt genome sequences obtained in the present work. Additionally, the presence of five single nucleotide polymorphisms (SNPs) between the strains highlights a minor but consistent level of genetic variation. Previous results in *Eimeria* species shown that the mitochondrial genome is arranged in tandemly repeated linear 6.2 kb elements, usually contains a non-fragmented three protein-coding genes, with the transcriptional direction of Cytb, CoI and CoIII^66^. In contrast, *T. gondii* showed that fragmented cytochrome genes exist as nonrandom concatemers in the mitochondrial genome, similar to the organization seen in related Coccidians, *Hammondia* and *Neospora*. For *N. caninum*, 20 distinct contigs ranging from 1.4 to 86 kb were identified, while *T. gondii* had 29 contigs ranging from 1.1 to 39 kb^29,30^ *Cystoisospora (Isospora)* was initially classified within the family Eimeriidae alongside *Eimeria* and *Cyclospora* spp., due to its homoxenous life cycle that resembles that of *Eimeria* species^67^. However, genetic reevaluations of coccidian phylogeny have repositioned *C. suis* as an outgroup within the family Sarcocystidae, distinct from the cluster comprising the genera *Neospora*,* Hammondia*, and *Toxoplasma*^31,68^. Notably, *Cystoisospora* spp. are most closely related to *T. gondii* and *N. caninum*. Additionally, the nearest outgroup family to Sarcocystidae is Eimeridae^69^. The mitochondrial genome sequences indicate that the piglet coccidia is related to other members of their family and closely related to *Eimeria* species. Our results suggest that *C. suis* has a mitochondrial structure similar to that of its close relatives, positioning it as an intermediate example within the coccidians.

Toltrazuril, like other triazine anticoccidials, exerts its effects through various mechanisms, including interference with the mitochondrial respiratory chain. This interference may lead to reduced enzymatic activity within the respiratory chain, contributing to its efficacy against Coccidia including *Eimeria tenella*^22,25,70^. In our study, qRT-PCR analysis provided key insights into the transcriptional regulation of mitochondrial genes in relation to toltrazuril susceptibility and action of the drug. Notably, the upregulation of these genes in Holland-I compared to Wien-I suggests increased baseline mitochondrial activity that could be associated with the parasite resistance phenotype. This might indicate an adaptive mechanism where *C. suis* strains that are less susceptible to toltrazuril potentially enhance their mitochondrial functions as a compensatory response to the presence of the drug. Further, the noticeable downregulation of CoI, CoIII, and Cytb mRNA levels in Holland-I following drug treatment underscores the mitochondrial genes responsiveness to the drug. This suggests that while these genes are upregulated in an untreated state, contributing to a possible resistance mechanism, they remain susceptible to drug action, indicating a complex interaction between the parasite genetic makeup and the drug mechanism of action. This dual behavior - upregulation in untreated conditions and downregulation upon treatment - shows the dynamic nature of gene expression in response to environmental stresses and pharmacological intervention. Similar mechanisms have been observed in other apicomplexans, where changes in mitochondrial gene expression and function contribute to the development of drug resistance^29^. The observed transcriptional changes are consistent with the proposed action of toltrazuril, which is based on disruption of the mitochondrial respiratory chain^22^ .

## Conclusions

Coccidian parasites demonstrate various resistance mechanisms, often involving genetic modifications that affect drug interaction pathways. The complex interplay of factors influencing drug resistance in *C. suis*, as observed in our study, points to the multifaceted nature of resistance mechanisms. These include not only genetic factors like SNPs and DEGs but also broader genomic adaptations involving mitochondrial functions and retrotransposon activities. Such complexity suggests that resistance mechanisms in coccidian parasites are not solely dependent on one pathway or genetic change but result from a series of adaptations at various biological levels, suggesting multi-factorial resistance mechanisms that may be shared across the order of the Coccidia.

Given the intricate relationship between drug resistance and mitochondrial function observed in *C. suis*, future research should focus on mitochondrial targets for drug development. Understanding how mitochondrial adaptations contribute to resistance will enhance our ability to design drugs that can overcome or circumvent these adaptations. Moreover, expanding comparative genomic studies to include more coccidian species will help delineate common and unique adaptive strategies, ultimately informing more effective control measures against these parasites.

In conclusion, the comparative study of the *C. suis* strains Holland-I and Wien-I reveals significant genomic adaptations fundamental to understanding resistance mechanisms and the evolutionary pressures driving these changes. These insights not only highlight the genetic responses to pharmacological challenges but also reinforce the need for novel management strategies to combat parasite resistance effectively.

## Materials and methods

### *Cystoisospora suis* oocyst collection

Oocysts of strains Wien-I (toltrazuril susceptible^71^;) or Holland-I (toltrazuril resistant^6^;) were purified from the faeces of experimentally infected piglets (according to §§ 26 ff. of the Animal Experiments Act, Tierversuchsgesetz 2021—TVG 2012 under number 2021–0.030.760.; Austrian Federal Ministry of Science, Health and Economy) using 25% Percoll (GE Healthcare, Vienna, Austria), washed and sporulated as described^72^.

In vitro culture.

Intestinal porcine epithelial cells (IPEC-1, ACC 705, Leibniz Institute DSMZ-German Collection of Microorganisms and Cell Cultures GmbH, Leibniz, Germany) were used as host cells in vitro and seeded in a density of 4 × 10^5^ cells per well in a 6-well plate (VWR, Vienna, Austria). Cells were grown in in DMEM/Ham’s F-12 medium (Gibco-Fisher Scientific GmbH, Schwerte, Germany) with 5% foetal calf serum (Gibco) and 100 U/ml penicillin and 0.1 mg/ml streptomycin (PAN- biotech GmbH, Aidenbach Germany) at 37 °C in 5% CO_2_. Confluent IPEC-1 cells were infected with 5 × 10^3^ sporozoites/well released from excysted oocysts and incubated further at 40 °C under 5% CO_2_. Infected cells with Holland-I were additionally incubated in the presence of 10 µM of toltrazuril (Sigma-Aldrich, Misuri, USA) dissolved in DMSO from day 6 for 72 h as described^32^. Parasites were counted daily to follow the growth patterns of the two strains used (supplemental data 2).

### DNA and RNA extraction and Illumina sequencing

DNA was extracted from seven biological replicates of dependent samples by well-wise pooling the cell culture supernatant containing free merozoites seven to nine days after infection of cells. Extraction was carried out using PeqGold Microspin Tissue DNA kit (VWR, Vienna, Austria) following the manufacturer’s instructions. The elution volume was 100 µl of Mili-Q water. The DNA samples were stored at − 20 °C until use. Libraries were prepared using the NEBNext Ultra II DNA Library Prep Kit (E7645L, New England Biolabs, Ipswich, MA) and sequenced on a Illumina NovaSeq S4 using a 2 × 150 bp paired-end protocol.

RNA was isolated using RNeasy Mini kit (Qiagen, Hilden, Germany) and treated with RNase-free DNase (Qiagen) according to the manufacturer’s instructions to remove any DNA contamination. Total RNA was quantified using a NanoDrop 2000 (Thermo Fischer Scientific, Waltham, MA, USA). RNA samples with an RNA integrity number above 8.0 were used for library preparation, and samples were sent for library preparation using a reverse stranded protocol with poly-A enrichment. Sequencing libraries were prepared using the NEBNext Poly(A) mRNA Magnetic Isolation Module and the NEBNext Ultra II Directional RNA Library Prep Kit for Illumina according to manufacturer’s protocols (New England Biolabs, Ipswich, Massachusetts, USA). Libraries were QC-checked on a Bioanalyzer 2100 (Agilent Technologies, Santa Clara, CA, USA) using a High Sensitivity DNA kit for correct insert size and quantified using Qubit dsDNA HS assay (Invitrogen, Waltham, Massachusetts, USA). Pooled libraries were sequenced on a NextSeq2000 instrument (Illumina, San Diego, California, USA) in 2 × 150 bp paired-end sequencing mode.

Sequence data that support the findings of this study have been deposited in the Sequence Read Archive (SRA) under the accession numbers SRR29155957 and SRR29155956. The RNA-seq data have been deposited in the Gene Expression Omnibus (GEO) under the accession number GSE268275.

### Bioinformatic processing of the RNA-seq data

The raw data underwent preprocessing using PRINSEQ-lite^73^ (version 0.20.4), followed by alignment of the remaining high-quality reads to the *C. suis* reference genome (GenBank assembly accession: GCA_002600585.1, Genome assembly: ASM260058v1) using STAR^74^(version 2.7.9a). Alignment processing was performed using samtools^75^(version 1.4). Subsequently, featureCounts^76^(version 2.0.3) from the Subread package was employed to quantify reads per gene. Differential gene expression analysis was then conducted using DESeq2^77^. Heatmaps were generated using the heatmap.2 function from the gplots R package^78^.

### Bioinformatic processing of the WGS data

The preprocessing of raw reads was conducted using PRINSEQ-lite^79^ (version 0.20.4). Subsequently, alignment against the *Sus scrofa* reference sequence (Sscrofa11.1 GCA_000003025.6) was performed using BWA (version 0.7.17-r1188). Post-processing and segregation into swine and non-swine reads were carried out using samtools^79^ (version 1.4). Paired non-swine reads were isolated using PRINSEQ-lite.

Following this, alignment to the *C. suis* reference genome was executed with BWA, with subsequent post-processing utilizing samtools. Additional steps included the addition of read groups and duplicate marking using Picard^80^ (version 3.1.1).

For variant calling, HaplotypeCaller, CombineGVCFs, and FilterVcf from the GATK package^81^(version 4.5.0) were utilized to apply BaseRecalibrator via ApplyBQSR. SNPs and short InDels were then called using freebayes^82^(version 1.3.6). Post-variant calling, VCFtools^83^(version 0.1.16) was employed for filtering, and bcftools^84^(version 1.19) was used to separate variants specific to Holland-I and Wien-I strains. Normalization of the read coverage to CPM was done with bamCoverage from deeptools^85^ and for the circular representation of genes and variants the circlize R package^86^ was utilized.

### Mitochondria PCR amplification and sequencing

Initially, Primers were designed from highly conserved regions of available mitochondrial genome sequences for *Eimeria* species and based on the partial mitochondrial sequences of *T. gondii*. Three pairs of primers were designed in the conserved regions of the partial COIII and cytb to amplify three fragments (Table S4). Mitochondrial DNA fragments were amplified by PCR from cDNA using a Q5 high Fidelity DNA Polymerase (New England Biolabs, Ipswich, Massachusetts, USA) The cycling conditions were: 95 °C for 2 min (initial denaturation), then 95 °C for 30 s (denaturation), 68 °C (PCR1) and 60ºC (PCR 2 and 3) for 30 s (annealing), and 72 °C for 2 min (extension) for 30 cycles, followed by 72 °C for 5 min (final extension). Each PCR reaction yielded a single band detected in a 1% agarose gel stained with Midori Green Advance (Nippon Genetics Europe, Düren, Germany). DNA bands were excised from the gel and purified using a QIA quick gel extraction and purification kit (Qiagen, Toronto ON, Canada) according to the manufacturer’s instruction.

Purified PCR products were sequenced in both directions using a primer-walking strategy to generate near–complete mitochondrial genomes essentially as described by Ogedengbe et al.^17^. Sequencing was carried out using the ABI 3730 XL Sequence Detection System (Applied Biosystem Inc., Foster City, CA, USA) with a read length up to 1,100 nt (PHRED20 quality) by LGC genomics GmbH (Berlin, Germany).

### qRT-PCR of mitochondrial genes

Synthesis of cDNA was accomplished using the iScript cDNA synthesis kit (Bio-Rad, Hercules, California, USA). Quantitative PCR amplification of cDNA was carried out on a Mx3000P thermal cycler (Agilent Technologies, Santa Clara, CA, USA). The primers for gene amplification are listed in Table S4. Reaction mixtures contained 2.5 µl of sample cDNA (50 ng/µl), 5 µl of SsoAdvance Universal Probes Supermix (Bio-Rad, Hercules, California, USA) and 1.3 µl of nuclease-free water with primers and probes at a final concentration of 500 and 200 nM, respectively. Activation of polymerase was performed at 95 °C for 2 min, followed by 40 cycles of 95 °C for 15 s and 60 °C for 30 s. Each sample was run in triplicate. The qPCR results were normalized against the mean of two reference genes, GAPDH and actin (see primers in Table S4). Average gene expression relative to the endogenous control for each sample was calculated using the 2 − ΔΔCq method. The relative fold change of gene expression was expressed as the mean and standard deviation. Statistical analyses were performed using the ANOVA one way test with the software GraphPad Prism 10.2.2 (GraphPad Software, San Diego, CA). Differences were considered statistically significant at *P* ≤ 0.05.

### Gene annotation analyses

A consensus sequence was constructed by alignment of the forward and reverse sequences using the Assembly contigs tools of SnapGene (Version 6.2.1).

Gene annotations available on www.toxodb.org were used for *C. suis* data described in this study. The identification of potential homologues of *C. suis* hypothetical genes was also carried out using a BLAST analyses on www.toxodb.org and https://blast.ncbi.nlm.nih.gov/blast/Blast.cgi.

## Electronic supplementary material

Below is the link to the electronic supplementary material.

Supplementary Material 1

Supplementary Material 2

Supplementary Material 3

Supplementary Material 4

Supplementary Material 5

Supplementary Material 6

## Data Availability

Sequence data that support the findings of this study have been deposited in the Sequence Read Archive (SRA) under the accession numbers SRR29155957 and SRR29155956. The RNA-seq data have been deposited in the Gene Expression Omnibus (GEO) under the accession number GSE268275.
