# Supplementary material for: Deciphering the code of resistance: a genomic and transcriptomic exploration of the Cystoisospora suis Holland-I strain
Source: Sci Rep. 2025 Feb 14;15:5461. doi: 10.1038/s41598-025-89372-8 (PMC11828913; doi:10.1038/s41598-025-89372-8)

**Table 1**. Primers used in this work

| N | Primer | Sequences (5’→ 3’) | Amplicon length (bp) |
| --- | --- | --- | --- |
|  | **For sequencing** |  |  |
| 1 | Fw-primer 1 | CGTAAGGAAAAGGAAAGGTTAACCGCTGT |  |
| 2 | Rv-primer 2 | CCCGCAGGATTACTAGAACCGTTTAGATG | 3068 PCR 1 |
| 3 | Fw-primer 3 | TCTATCGGTAAAAAGGTACGCCGGGGATAACA |  |
| 4 | Rv- primer 4 | AGTAGGGGCTATCATTTGGTTCTATACTC | 868 PCR 2 |
| 5 | Fw-primer 5 | CCTTACTTAGTACCTTGGTTACTAGGAGGG |  |
| 6 | Rv-primer-6 | CGGTAACTGTTGTGTTTAGATAGCGG | 1962 PCR 3 |
| 7 | Fw-int-1 | GGGTCCATGTACAATTACTAGCTGAGA |  |
| 8 | Rv-int-2 | CCTAAATTCCCATCCAGCACCTACTTC |  |
| 9 | Fw-int-3 | CTGTTATTTACCTTAGGAGGTACTACAGGTGTAG |  |
| 10 | Rv-int-4 | GCACCTAAAGATAACACAAAATGGAAGTGAGCT |  |
| 11 | Fw-int-5 | GCGTACAACATATAGTTAGGGAAGTAGGTGC |  |
| 12 | Fw-int-6 | CCGGTGGTTTGTTAGTATTTATGTCATCATTG |  |
| 13 | Fw-int-7 | TCTTGGATGATTGTATATTAGCGGCTAAATGTC |  |
| 14 | Fw-int-8 | GGTCATAAGACTAGTACTTAGAGGCGGATAC |  |
| 15 | Rv-int-9 | CGTATTACATCTTACGGTGAACTATCGTTCCT |  |
| 16 | Rv-int-10 | CTTGGGTACTATAATGTCTAATATGATCCGTATCG |  |
|  | **For RtqPCR** |  |  |
| 1 | Fw-ACTIN | CTTGCTGGCCGTGATTTGAC |  |
| 2 | Rv-ACTIN | ATATTGCCGTCCGGAAGCTC | 203 |
| 3 | Probe-ACTIN | CCTCCGCCGAGAAGGAAATT |  |
| 4 | Fw-GAPDH | TTCAACGAGAAGGAGCCAAG |  |
| 5 | Rv-GAPDH | CTTCGGAGGTGCAGACATG | 150 |
| 6 | Probe-GAPDH | CAAGGAAAAGGCTGAGGCGCAT |  |
| 7 | Fw-CoI | GATACATTGTCCAACCCATACC |  |
| 8 | Rv-CoI | GTTCCCTCGAATTAACGCAG | 110 |
| 9 | Probe-CoI | CCGAACTCGGAGGTTATACTCTGAATAACAA |  |
| 10 | Fw-CoIII | GGGTCCATGTACAATTACTAGCTGAGA |  |
| 11 | Rv-CoIII | CTCCTTAGAGGGAGTCTGTG | 158 |
| 12 | Probe-CoIII | TTGATACGACGACACTTAATGCAGACAATAG |  |
| 13 | Fw-CytB | TTTAACTACTGCCTGGTTGTC |  |
| 14 | Rv- CytB | TCCCCAAAAACTCATTTGTCC | 123 |
| 15 | Probe- CytB | CTATTGCAACTGCCTTTCTTGGATATGTGTTA |  |


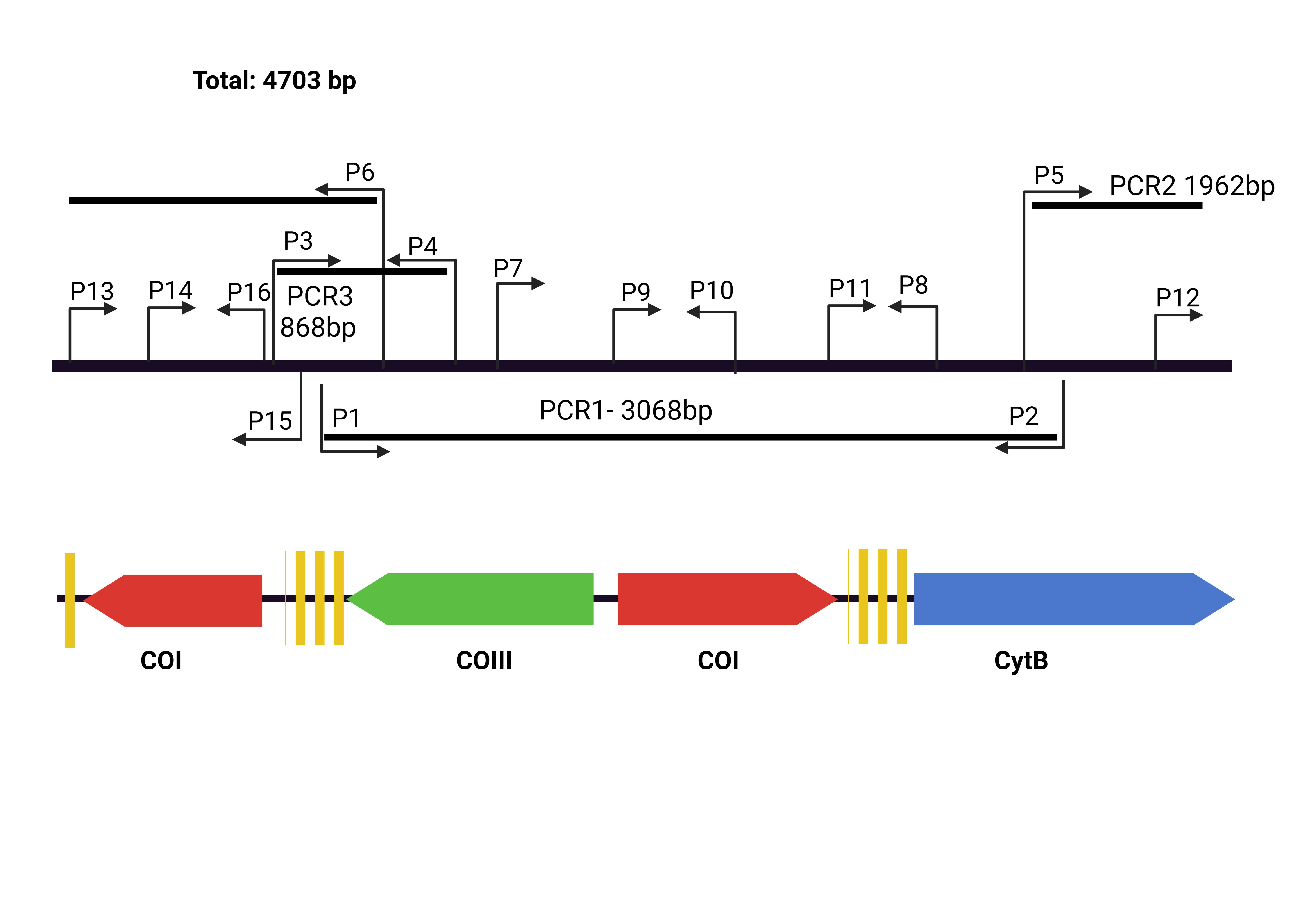

Supplement: Supplementary file 4 — Supplementary Material 4 [file 41598_2025_89372_MOESM4_ESM.docx]
