# Supplementary material for: Deciphering the code of resistance: a genomic and transcriptomic exploration of the Cystoisospora suis Holland-I strain
Source: Sci Rep. 2025 Feb 14;15:5461. doi: 10.1038/s41598-025-89372-8 (PMC11828913; doi:10.1038/s41598-025-89372-8)
Supplement: Supplementary file 5 — Supplementary Material 5 [file 41598_2025_89372_MOESM5_ESM.docx]

Data set-Wien-I

GTATTGATACTCCGCTTAAAGTGCCTTTTTATCCTCATATGCTAATGACTGATGGAAAAGGACTAAGTTATTTACTTGTGTTAGTTTTCCTACAAGCGGCTTTTGGTTGGATCGAATTATCCCATCCAGACAATTCAATACCAGTAAATCGTTTTGTAACTCCGCTACATATAGTCCCCGAATGGTATTTCTTAGCCTACTATGCGGTGTTAAAAGTCATTCCAAGTAAAACCGGTGGTTTGTTAGTATTTATGTCATCATTGATAATGAAATAGTAAATGAAACAAATGTAAAAACATAACATAATAAAAGGTTGTGACTACTAAGTTCATTTGGTTATGTTTTGAACACAAACCATTGATAATACGAAGTTATGGTTTTGGGCTCGTTTGTGTCTCTCAATAACTAGCTGAGTGCTTGTGCGATAATTATATCAATGCTAAATTATATAGTTTTTTCAGGAGCATACCGTGAATATCGATGATCTTTGAATGACTACAGCTTCCAAGCATACATTGTATAGTCTGATCTCAAAGTACCAGAAGACATGTGATCTATATAATATAACCGGACTTTTGACCGAACCTGCGATAGATAAATATATCTTGGATGATTGTATATTAGCGGCTAAATGTCAATCAAACATGATAATTTTAGGTTTTCCATGAAATCTATTTGGAAGAAGAGGCTTGATAGTACAACCGTAAGTACAGAAGATACAGTCCCAGCAGTAGCGGTCAAACTATAGAAGAGTCGAGTATTATCCATGCATACCAGGCGTAAAAAGCGTTCATCCAGATACTAAACAGGTGCCAGGCCAACAATGATCCAATCAGTGGATTCCGTATTCAAGGTCATAAGACTAGTACTTAGAGGCGGATACATTGTCCAACCCATACCACTACCGAACTCGGAGGTTATACTCTGAATAACAAATATAGAACCTAAAGGTACTAGGAAGTAAGATACTGCGTTAATTCGAGGGAACACTACTTCTGAAGCCCCTATGTATATAGGTAGAAAGAAGTTACCATATCCCCCGTACAGAGCAGGGATTAAGAACATAAAGATCATAGCTAGACCATGTATCGTAATAATAACGTTATATGTAGAAATGGTTTCTGCACAAATAATTCGGGAACCGCTATTATAAAGTTCGATACGGATCATATTAGACATTATAGTACCCAAGACACTGAATATAGCTCCAGTAACTAGGTACATAGCTCCTATTTCTTTATGATTATTATATGCTAGAGGGTACACTTCATCAAATAACGGTACAAAGGTACGCCGGGGATAACAGGTCAGATAAAATCGGGAGTTCATATCCTCGGATTATATCAGCACCTCCATGTCGGCTCATTACTCCCTTGTTGTTGAACAATATTCAATTAGGAACGATAGTTCACCGTAAGATGTAATACGTGAGCTGGGTTAAGAACGTCTTGAGACAGTTTGTTCCCTATCTACCATACATAATAGTAACTGTCCTAACTTGGCATAACGTTGTGTAGTTAGTAGGAGCGTACATTCCTTAATATCTGGAACAATGGATTAAGTTTAGGTAGTGGAACAAGGAGTCTAGCTTCAGATGTTATCTGATGTTGTATGCCCTGAGTACGTAAGGAAAAGGAAAGGTTAACCGCTATCTAAACACAACAGTTACCGTAGCTGTAGATAAATGCTATATCAAGAGTGTATCTACTAAGATACTGCATAACATATAAATGCTCCTACCGCCATCCGTTGACTGTGTTTTCCACGGGGAATTAGAACTGAAATCAACATGGAAGCTGGAAGACGGAATCGTTCTTAAGCGCCAGGTAGTCCTGGGCACTGAATCCATGAGCGTGACTTTATGGATACGTTTTTGTTAGACGCTAACTTCCCGGCTAAACATCCCTTTTCTTTGAAACACACTTCCCTTCTCGCCGTAAGTATATTTATAGATAGATTGTCACATGGATAGCTAACCAGAGACTTTCTACGAAATGCAGATATAGAATTGTAAAAGGTTCAGTTTTTAGAGCTAAGAATGTAGATTGTTGGAATTGTAAACCAGAGTTATTTACAGGAACATAAGTGGTAAAACTATTGTAAGGTGATTGAGTATAGAACCAAATGATAGCCCCTACTATAACATGACTAAAATGTAAACCTGTAAGCACAAACATTACATTACCCATAGCACTATCGTTAATATAAAAAGATAGTCCTAAATATTCGCTGATTAGTAGTACGCAGAAGAGTTCGACTAACCAAAGGGTCCATGTACAATTACTAGCTGAGATCATATTCATATTTTTCAAGTATTGGCTTGATACGACGACACTTAATGCAGACAATAGAATGGTCATGAGTATAACAATAGAGCTAGCACTAGGTGCACAGACTCCCTCTAAGGAGAGACCGGAGGTCCATCCCGTACTATATGCCGCCCAGAAGAAGCTTATAAATAACCCTGTCTCGGATAATATTACCCCTAGTACAATAGTACTAATAAGACTAGTATCCGAATAGTATATTTCCCTTGTAGCAAATAATATAATCATAAATGTTCCATATACCAATGGATTTAATACCCAACCAATATTAAATAACCTTAAAGTCGTAGGATATTGATAGACCATGCTTTTATATGTACAAAGTTTACTACACAACCTTAATGCAGTGAGGGACATAATTAATCCTTGTACGGTTCGTACCTACTTGACTCCTCAGTTTAAGCTGAATTGTAGTTTATCGGGACTAAAGTCAGCATAGTCAATTAAAAGGTTTGTTCTCGTAACCATGCTAACACAAATGACATATATATATACATTCCGCATTAATTGTGGTTATATAAATCTGTTAATGCTTGTCAAGTTCCTTTTATATAGCTATCTCACAGCGTACTTAGGTTCCAACTAAACGAGTTCTCTGTTGGTGTTATATCACAGACTCTGTCTACATATGCTACCCGAACGGTATTCGGTGGTCAATCTATGATCTTAGCTATGGGTTGTATATCTATTCTAGGTGGATTGGTTTGGGCACATCATATGATGACAGTAGGATTAGAAGTAGATACTCGTGCATATTTCTCTGCTGTAACGATTATGATTGCAATACCTACTGGTACTAAAATATTCAATTGGCTTGGTACTTATATGGCAAGTAACTATGCTACTAGGAATGTAGATTTATGGGCGGCTCTTAGCTTTATTCTGTTATTTACCTTAGGAGGTACTACAGGTGTAGTTATGGGTAATGCAGCTATGGATATAGCATTACACGATACATATTATATTGTAGCTCACTTCCATTTTGTGTTATCTTTAGGTGCTGTATTAGCTACAATTTGTGGTTTTGTATATTATGCTAAAGATATGTTTGGAGATACTTTAAACTTCTTCTTACCTGTGACAGGTGGTTCACATTTCCTAAGTATTTGGTTCTTTGTATTCCTATGTAGTATTATGCTTATCTTCATACCTATGCATATATTAGGTTTTAATGTTATGCCTCGAAGGATACCAGATTATCCCGATTTTATTACTTATATTAACACAATGTGTTCGATTGGTTCAATCTCTACTATCCTAATCATCTTAGCTATGCTTTTATAATACAATAAGATTACAGGCGCATGTAAACTAGTCTTAAACACACCGCTCGTCACGTAAAAGAATCAATGGTGAGTCCTTTTGGTTGCTCCTTCTGGCTAGACTTTGACTTGTTCAACCAGCCTGGGATCACAAAATATCTTTGTATGGTAAGATTGAGCGTGGACTATCGAATGAAACAATGTGCTCCACCGCTAGTCTAAGTCTCTAACATACCTTTTACCTTCAACTGTACGGAACGTAACAAACCTCCAGGCAAAGAATAATATTTTGTTCAGCCACTGGTTCACCGTCAACTACCTTGTTTCGACTTCGTACCGACTGTGTTATTGTAGCACATACTCTACCCTATGAAAGGGAGTCTATTCCCAAACAACCGGATCGTGTTGGCTAGGTGAACTAATCACGTTTCATAAATACAATCAGTGAAAGCTCTTTTGATTTCCATGAAGTGAGTTACATATTAGATTCTTTTCGCTCCCTAAGAGTACCGCACCTATGAATATATGACACTCGTACGAGCACATCTAGTCTATTACAGATGTGCTTTAAATATCAATTTTTCATATAACTTTGGTTTCTTACTTGCTATTGCCTTTGTAATGCAAATTATTACTGGTATTACTTTAGCCCTTCGATATACCTCGGAAGCTTCATACGCCTTTGTTAGCGTACAACATATAGTTAGGGAAGTAGGTGCTGGATGGGAATTTAGGATGTTACATGCTACTACTGCCTCATGTGTATTCGCGTTTATTTTAGTTCATATGATTCGAGGAATGTACAACAGTAGTTACAGCTATTTAACTACTGCCTGGTTGTCTGGCTTGGTTTTATATATTATTACTATTGCAACTGCCTTTCTTGGATATGTGTTACCATGGGGACAAATGAGTTTTTGGGGAGCCACAGTCATCACCAACCTCTTGTCTCCAATCCCTTACTTAGTACCTTGGTTACTAGGAGGGTTTTATGTCTCTGATGTTACCTTAAAACGTTTCTTCGTTTTACATTTTGTACTTCCTTTTGTAGGTTGTGTTCTAATAGTATTACATATCTTCTATTTACATCTAAACGGTTCTAGTATCCCTGCGG

Data set Holland-I strains

GTATTGATACTCCGCTTAAAGTGCCTTTTTATCCTCATATGCTAATGACTGATGGAAAAGGACTAAGTTATTTACTTGTGTTAGTTTTCCTACAAGCGGCTTTTGGTTGGATCGAATTATCCCATCCAGACAATTCAATACCAGTAAATCGTTTTGTAACTCCGCTACATATAGTCCCCGAATGGTATTTCTTAGCCTACTATGCGGTGTTAAAAGTCATTCCAAGTAAAACCGGTGGTTTGTTAGTATTTATGTCATCATTGATAATGAAATAGTAAATGAAACAAATGTAAAAACATAACATAATAAAAGGTTGTGACTACTAAGTTCATTTGGTTATGTTTTGAACACAAACCATTGATAATACGAAGTTATGGTTTTGGGCTCGTTTGTGTCTCTCAATAACTAGCTGAGTGCTTGTGCGATAATTATATCAATGCTAAATTATATAGTTTTTTCAGGAGCATACCGTGAATATCGATGATCTTTGAATGACTACAGCTTCCAAGCATACATTGTATAGTCTGATCTCAAAGTACCAGAAGACATGTGATCTATATAATATAACCGGACTTTTGACCGAACCTGCGATAGATAAATATATCTTGGATGATTGTATATTAGCGGCTAAATGTCAATCAAACATGATAATTTTAGGTTTTCCATGAAATCTATTTGGAAGAAGAGGCTTGATAGTACAACCGTAAGTACAGAAGATACAGTCCCAGCAGTAGCGGTCAAACTATAGAAGAGTCGAGTATTATCCATGCATACCAGGCGTAAAAAGCGTTCATCCAGATACTAAACAGGTGCCAGGCCAACAATGATCCAATCAGTGGATTCCGTATTCAAGGTCATAAGACTAGTACTTAGAGGCGGATACATTGTCCAACCCATACCACTACCGAACTCGGAGGTTATACTCTGAATAACAAATATAGAACCTAAAGGTACTAGGAAGTAAGATACTGCGTTAATTCGAGGGAACACTACTTCTGAAGCCCCTATGTATATAGGTAGAAAGAAGTTACCATATCCCCCGTACAGAGCAGGCATTAAGAACATAAAGATCATAGCTAGACCATGTATCGTAATAATAACGTTATATGTAGAAATCGTTTCTGCACAAATAATTCGGGAACCGCTATTATAAAGTTCGATACGGATCATATTAGACATTATAGTACCCAAGACACTGAATATAGCTCCAGTAACTAGGTACATAGCTCCTATTTCTTTATGATTATTATATGCTAGAGGGTACACTTCATCAAATAACGGTACAAAGGTACGCCGGGGATAACAGGTCAGATAAAATCGGGAGTTCATATCCTCGGATTATATCAGCACCTCCATGTCGGCTCATTACTCCCTTGTTGTTGAACAATATTCAATTAGGAACGATAGTTCACCGTAAGATGTAATACGTGAGCTGGGTTAAGAACGTCTTGAGACAGTTTGTTCCCTATCTACCATACATAATAGTAACTGTCCTAACTTGGCATAACGTTGTGTAGTTAGTAGGAGCGTACATTCCTTAATATCTGGAACAATGGATTAAGTTTAGGTAGTGGAACAAGGAGTCTAGCTTCAGATGTTATCTGATGTTGTATGCCCTGAGTACGTAAGGAAAAGGAAAGGTTAACCGCTGTCTAAACACAACAGTTACCGTAGCTGTAGATAAATGCTATATCAAGAGTGTATCTACTAAGATACTGCATAACATATAAATGCTCCTACCGCCATCCGTTGACTGTGTTTTCCACGGGGAATTAGAACTGAAATCAACATGGAAGCTGGAAGACGGAATCGTTCTTAAGCGCCAGGTAGTCCTGGGCACTGAATCCATGAGCGTGACTTTATGGATACGTTTTTGTTAGACGCTAACTTCCCGGCTAAACATCCCTTTTCTTTGAAACACACTTCCCTTCTCGCCGTAAGTATATTTATAGATAGATTGTCACATGGATAGCTAACCAGAGACTTTCTACGAAATGCAGATATAGAATTGTAAAAGGTTCAGTTTTTAGAGCTAAGAATGTAGATTGTTGGAATTGTAAACCAGAGTTATTTACAGGAACATAAGTGGTAAAACTATTGTAAGGTGATTGAGTATAGAACCAAATGATAGCCCCTACTATAACATGACTAAAATGTAAACCTGTAAGCACAAACATTACATTACCCATAGCACTATCGTTAATATAAAAAGATAGTCCTAAATATTCGCTGATTAGTAGTACGCAGAAGAGTTCGACTAACCAAAGGGTCCATGTACAATTACTAGCTGAGATCATATTCATATTTTTTAAGTATTGGCTTGATACGACGACACTTAATGCAGACAATAGAATGGTCATGAGTATAACAATAGAGCTAGCACTAGGTGCACAGACTCCCTCTAAGGAGAGACCGGAGGTCCATCCCGTACTATATGCCGCCCAGAAGAAGCTTATAAATAACCCTGTCTCGGATAATATTACCCCTAGTACAATAGTACTAATAAGACTAGTATCCGAATAGTATATTTCCCTTGTAGCAAATAATATAATCATAAATGTTCCATATACCAATGGATTTAATACCCAACCAATATTAAATAACCTTAAAGTCGTAGGATATTGATAGACCATGCTTTTATATGTACAAAGTTTACTACACAACCTTAATGCAGTGAGGGACATAATTAATCCTTGTACGGTTCGTACCTACTTGACTCCTCAGTTTAAGCTGAATTGTAGTTTATCGGGACTAAAGTCAGCATAGTCAATTAAAAGGTTTGTTCTCGTAACCATGCTAACACAAATGACATATATATATACATTCCGCATTAATTGTGGTTATATAAATCTGTTAATGCTTGTCAAGTTCCTTTTATATAGCTATCTCACAGCGTACTTAGGTTCCAACTAAACGAGTTCTCTGTTGGTGTTATATCACAGACTCTGTCTACATATGCTACCCGAACGGTATTCGGTGGTCAATCTATGATCTTAGCTATGGGTTGTATATCTATTCTAGGTGGATTGGTTTGGGCACATCATATGATGACAGTAGGATTAGAAGTAGATACTCGTGCATATTTCTCTGCTGTAACGATTATGATTGCAATACCTACTGGTACTAAAATATTCAATTGGCTTGGTACTTATATGGCAAGTAACTATGCTACTAGGAATGTAGATTTATGGGCGGCTCTTAGCTTTATTCTGTTATTTACCTTAGGAGGTACTACAGGTGTAGTTATGGGTAATGCAGCTATGGATATAGCATTACACGATACATATTATATTGTAGCTCACTTCCATTTTGTGTTATCTTTAGGTGCTGTATTAGCTACAATTTGTGGTTTTGTATATTATGCTAAAGATATGTTTGGAGATACTTTAAACTTCTTCTTACCTGTGACAGGTGGTTCACATTTCCTAAGTATTTGGTTCTTTGTATTCCTATGTAGTATTATGCTTATCTTCATACCTATGCATATATTAGGTTTTAATGTTATGCCTCGAAGGATACCAGATTATCCCGATTTTATTACTTATATTAACACAATGTGTTCGATTGGTTCAATCTCTACTATCCTAATCATCTTAGCTATGCTTTTATAATACAATAAGATTACAGGCGCATGTAAACTAGTCTTAAACACACCGCTCGTCACGTAAAAGAATCAATGGTGAGTCCTTTTGGTTGCTCCTTCTGGCTAGACTTTGACTTGTTCAACCAGCCTGGGATCACAAAATATCTTTGTATGGTAAGATTGAGCGTGGACTATCGAATGAAACAATGTGCTCCACCGCTAGTCTAAGTCTCTAACATACCTTTTACCTTCAACTGTACGGAACGTAACAAACCTCCAGGCAAAGAATAATATTTTGTTCAGCCACTGGTTCACCGTCAACTACCTTGTTTCGACTTCGTACCGACTGTGTTATTGTAGCACATACTCTACCCTATGAAAGGGAGTCTATTCCCAAACAACCGGATCGTGTTGGCTAGGTGAACTAATCACGTTTCATAAATACAATCAGTGAAAGCTCTTTTGATTTCCATGAAGTGAGTTACATATTAGATTCTTTTCGCTCCCTAAGAGTACCGCACCTATGAATATATGACACTCGTACGAGCACATCTAGTCTATTACAGATGTGCTTTAAATATCAATTTTTCATATAACTTTGGTTTCTTACTTGCTATTGCCTTTGTAATGCAAATTATTACTGGTATTACTTTAGCCCTTCGATATACCTCGGAAGCTTCATACGCCTTTGTTAGCGTACAACATATAGTTAGGGAAGTAGGTGCTGGATGGGAATTTAGGATGTTACATGCTACTACTGCCTCATGTGTATTCGCGTTTATTTTAGTTCATATGATTCGAGGAATGTACAACAGTAGTTACAGCTATTTAACTACTGCCTGGTTGTCTGGCTTGGTTTTATATATTATTACTATTGCAACTGCCTTTCTTGGATATGTGTTACCATGGGGACAAATGAGTTTTTGGGGAGCCACAGTCATCACCAACCTCTTGTCTCCAATCCCTTACTTAGTACCTTGGTTACTAGGAGGGTTTTATGTCTCTGATGTTACCTTAAAACGTTTCTTCGTTTTACATTTTGTACTTCCTTTTGTAGGTTGTGTTCTAATAGTATTACATATCTTCTATTTACATCTAAACGGTTCTAGTAATCCTGCGG

CLUSTAL O(1.2.4) multiple sequence alignment

Wien-I GTATTGATACTCCGCTTAAAGTGCCTTTTTATCCTCATATGCTAATGACTGATGGAAAAG 60

Hol-I GTATTGATACTCCGCTTAAAGTGCCTTTTTATCCTCATATGCTAATGACTGATGGAAAAG 60

************************************************************

Wien-I GACTAAGTTATTTACTTGTGTTAGTTTTCCTACAAGCGGCTTTTGGTTGGATCGAATTAT 120

Hol-I GACTAAGTTATTTACTTGTGTTAGTTTTCCTACAAGCGGCTTTTGGTTGGATCGAATTAT 120

************************************************************

Wien-I CCCATCCAGACAATTCAATACCAGTAAATCGTTTTGTAACTCCGCTACATATAGTCCCCG 180

Hol-I CCCATCCAGACAATTCAATACCAGTAAATCGTTTTGTAACTCCGCTACATATAGTCCCCG 180

************************************************************

Wien-I AATGGTATTTCTTAGCCTACTATGCGGTGTTAAAAGTCATTCCAAGTAAAACCGGTGGTT 240

Hol-I AATGGTATTTCTTAGCCTACTATGCGGTGTTAAAAGTCATTCCAAGTAAAACCGGTGGTT 240

************************************************************

Wien-I TGTTAGTATTTATGTCATCATTGATAATGAAATAGTAAATGAAACAAATGTAAAAACATA 300

Hol-I TGTTAGTATTTATGTCATCATTGATAATGAAATAGTAAATGAAACAAATGTAAAAACATA 300

************************************************************

Wien-I ACATAATAAAAGGTTGTGACTACTAAGTTCATTTGGTTATGTTTTGAACACAAACCATTG 360

Hol-I ACATAATAAAAGGTTGTGACTACTAAGTTCATTTGGTTATGTTTTGAACACAAACCATTG 360

************************************************************

Wien-I ATAATACGAAGTTATGGTTTTGGGCTCGTTTGTGTCTCTCAATAACTAGCTGAGTGCTTG 420

Hol-I ATAATACGAAGTTATGGTTTTGGGCTCGTTTGTGTCTCTCAATAACTAGCTGAGTGCTTG 420

************************************************************

Wien-I TGCGATAATTATATCAATGCTAAATTATATAGTTTTTTCAGGAGCATACCGTGAATATCG 480

Hol-I TGCGATAATTATATCAATGCTAAATTATATAGTTTTTTCAGGAGCATACCGTGAATATCG 480

************************************************************

Wien-I ATGATCTTTGAATGACTACAGCTTCCAAGCATACATTGTATAGTCTGATCTCAAAGTACC 540

Hol-I ATGATCTTTGAATGACTACAGCTTCCAAGCATACATTGTATAGTCTGATCTCAAAGTACC 540

************************************************************

Wien-I AGAAGACATGTGATCTATATAATATAACCGGACTTTTGACCGAACCTGCGATAGATAAAT 600

Hol-I AGAAGACATGTGATCTATATAATATAACCGGACTTTTGACCGAACCTGCGATAGATAAAT 600

************************************************************

Wien-I ATATCTTGGATGATTGTATATTAGCGGCTAAATGTCAATCAAACATGATAATTTTAGGTT 660

Hol-I ATATCTTGGATGATTGTATATTAGCGGCTAAATGTCAATCAAACATGATAATTTTAGGTT 660

************************************************************

Wien-I TTCCATGAAATCTATTTGGAAGAAGAGGCTTGATAGTACAACCGTAAGTACAGAAGATAC 720

Hol-I TTCCATGAAATCTATTTGGAAGAAGAGGCTTGATAGTACAACCGTAAGTACAGAAGATAC 720

************************************************************

Wien-I AGTCCCAGCAGTAGCGGTCAAACTATAGAAGAGTCGAGTATTATCCATGCATACCAGGCG 780

Hol-I AGTCCCAGCAGTAGCGGTCAAACTATAGAAGAGTCGAGTATTATCCATGCATACCAGGCG 780

************************************************************

Wien-I TAAAAAGCGTTCATCCAGATACTAAACAGGTGCCAGGCCAACAATGATCCAATCAGTGGA 840

Hol-I TAAAAAGCGTTCATCCAGATACTAAACAGGTGCCAGGCCAACAATGATCCAATCAGTGGA 840

************************************************************

Wien-I TTCCGTATTCAAGGTCATAAGACTAGTACTTAGAGGCGGATACATTGTCCAACCCATACC 900

Hol-I TTCCGTATTCAAGGTCATAAGACTAGTACTTAGAGGCGGATACATTGTCCAACCCATACC 900

************************************************************

Wien-I ACTACCGAACTCGGAGGTTATACTCTGAATAACAAATATAGAACCTAAAGGTACTAGGAA 960

Hol-I ACTACCGAACTCGGAGGTTATACTCTGAATAACAAATATAGAACCTAAAGGTACTAGGAA 960

************************************************************

Wien-I GTAAGATACTGCGTTAATTCGAGGGAACACTACTTCTGAAGCCCCTATGTATATAGGTAG 1020

Hol-I GTAAGATACTGCGTTAATTCGAGGGAACACTACTTCTGAAGCCCCTATGTATATAGGTAG 1020

************************************************************

Wien-I AAAGAAGTTACCATATCCCCCGTACAGAGCAGGGATTAAGAACATAAAGATCATAGCTAG 1080

Hol-I AAAGAAGTTACCATATCCCCCGTACAGAGCAGGCATTAAGAACATAAAGATCATAGCTAG 1080

********************************* **************************

Wien-I ACCATGTATCGTAATAATAACGTTATATGTAGAAATGGTTTCTGCACAAATAATTCGGGA 1140

Hol-I ACCATGTATCGTAATAATAACGTTATATGTAGAAATCGTTTCTGCACAAATAATTCGGGA 1140

************************************ ***********************

Wien-I ACCGCTATTATAAAGTTCGATACGGATCATATTAGACATTATAGTACCCAAGACACTGAA 1200

Hol-I ACCGCTATTATAAAGTTCGATACGGATCATATTAGACATTATAGTACCCAAGACACTGAA 1200

************************************************************

Wien-I TATAGCTCCAGTAACTAGGTACATAGCTCCTATTTCTTTATGATTATTATATGCTAGAGG 1260

Hol-I TATAGCTCCAGTAACTAGGTACATAGCTCCTATTTCTTTATGATTATTATATGCTAGAGG 1260

************************************************************

Wien-I GTACACTTCATCAAATAACGGTACAAAGGTACGCCGGGGATAACAGGTCAGATAAAATCG 1320

Hol-I GTACACTTCATCAAATAACGGTACAAAGGTACGCCGGGGATAACAGGTCAGATAAAATCG 1320

************************************************************

Wien-I GGAGTTCATATCCTCGGATTATATCAGCACCTCCATGTCGGCTCATTACTCCCTTGTTGT 1380

Hol-I GGAGTTCATATCCTCGGATTATATCAGCACCTCCATGTCGGCTCATTACTCCCTTGTTGT 1380

************************************************************

Wien-I TGAACAATATTCAATTAGGAACGATAGTTCACCGTAAGATGTAATACGTGAGCTGGGTTA 1440

Hol-I TGAACAATATTCAATTAGGAACGATAGTTCACCGTAAGATGTAATACGTGAGCTGGGTTA 1440

************************************************************

Wien-I AGAACGTCTTGAGACAGTTTGTTCCCTATCTACCATACATAATAGTAACTGTCCTAACTT 1500

Hol-I AGAACGTCTTGAGACAGTTTGTTCCCTATCTACCATACATAATAGTAACTGTCCTAACTT 1500

************************************************************

Wien-I GGCATAACGTTGTGTAGTTAGTAGGAGCGTACATTCCTTAATATCTGGAACAATGGATTA 1560

Hol-I GGCATAACGTTGTGTAGTTAGTAGGAGCGTACATTCCTTAATATCTGGAACAATGGATTA 1560

************************************************************

Wien-I AGTTTAGGTAGTGGAACAAGGAGTCTAGCTTCAGATGTTATCTGATGTTGTATGCCCTGA 1620

Hol-I AGTTTAGGTAGTGGAACAAGGAGTCTAGCTTCAGATGTTATCTGATGTTGTATGCCCTGA 1620

************************************************************

Wien-I GTACGTAAGGAAAAGGAAAGGTTAACCGCTATCTAAACACAACAGTTACCGTAGCTGTAG 1680

Hol-I GTACGTAAGGAAAAGGAAAGGTTAACCGCTGTCTAAACACAACAGTTACCGTAGCTGTAG 1680

******************************.*****************************

Wien-I ATAAATGCTATATCAAGAGTGTATCTACTAAGATACTGCATAACATATAAATGCTCCTAC 1740

Hol-I ATAAATGCTATATCAAGAGTGTATCTACTAAGATACTGCATAACATATAAATGCTCCTAC 1740

************************************************************

Wien-I CGCCATCCGTTGACTGTGTTTTCCACGGGGAATTAGAACTGAAATCAACATGGAAGCTGG 1800

Hol-I CGCCATCCGTTGACTGTGTTTTCCACGGGGAATTAGAACTGAAATCAACATGGAAGCTGG 1800

************************************************************

Wien-I AAGACGGAATCGTTCTTAAGCGCCAGGTAGTCCTGGGCACTGAATCCATGAGCGTGACTT 1860

Hol-I AAGACGGAATCGTTCTTAAGCGCCAGGTAGTCCTGGGCACTGAATCCATGAGCGTGACTT 1860

************************************************************

Wien-I TATGGATACGTTTTTGTTAGACGCTAACTTCCCGGCTAAACATCCCTTTTCTTTGAAACA 1920

Hol-I TATGGATACGTTTTTGTTAGACGCTAACTTCCCGGCTAAACATCCCTTTTCTTTGAAACA 1920

************************************************************

Wien-I CACTTCCCTTCTCGCCGTAAGTATATTTATAGATAGATTGTCACATGGATAGCTAACCAG 1980

Hol-I CACTTCCCTTCTCGCCGTAAGTATATTTATAGATAGATTGTCACATGGATAGCTAACCAG 1980

************************************************************

Wien-I AGACTTTCTACGAAATGCAGATATAGAATTGTAAAAGGTTCAGTTTTTAGAGCTAAGAAT 2040

Hol-I AGACTTTCTACGAAATGCAGATATAGAATTGTAAAAGGTTCAGTTTTTAGAGCTAAGAAT 2040

************************************************************

Wien-I GTAGATTGTTGGAATTGTAAACCAGAGTTATTTACAGGAACATAAGTGGTAAAACTATTG 2100

Hol-I GTAGATTGTTGGAATTGTAAACCAGAGTTATTTACAGGAACATAAGTGGTAAAACTATTG 2100

************************************************************

Wien-I TAAGGTGATTGAGTATAGAACCAAATGATAGCCCCTACTATAACATGACTAAAATGTAAA 2160

Hol-I TAAGGTGATTGAGTATAGAACCAAATGATAGCCCCTACTATAACATGACTAAAATGTAAA 2160

************************************************************

Wien-I CCTGTAAGCACAAACATTACATTACCCATAGCACTATCGTTAATATAAAAAGATAGTCCT 2220

Hol-I CCTGTAAGCACAAACATTACATTACCCATAGCACTATCGTTAATATAAAAAGATAGTCCT 2220

************************************************************

Wien-I AAATATTCGCTGATTAGTAGTACGCAGAAGAGTTCGACTAACCAAAGGGTCCATGTACAA 2280

Hol-I AAATATTCGCTGATTAGTAGTACGCAGAAGAGTTCGACTAACCAAAGGGTCCATGTACAA 2280

************************************************************

Wien-I TTACTAGCTGAGATCATATTCATATTTTTCAAGTATTGGCTTGATACGACGACACTTAAT 2340

Hol-I TTACTAGCTGAGATCATATTCATATTTTTTAAGTATTGGCTTGATACGACGACACTTAAT 2340

***************************** ******************************

Wien-I GCAGACAATAGAATGGTCATGAGTATAACAATAGAGCTAGCACTAGGTGCACAGACTCCC 2400

Hol-I GCAGACAATAGAATGGTCATGAGTATAACAATAGAGCTAGCACTAGGTGCACAGACTCCC 2400

************************************************************

Wien-I TCTAAGGAGAGACCGGAGGTCCATCCCGTACTATATGCCGCCCAGAAGAAGCTTATAAAT 2460

Hol-I TCTAAGGAGAGACCGGAGGTCCATCCCGTACTATATGCCGCCCAGAAGAAGCTTATAAAT 2460

************************************************************

Wien-I AACCCTGTCTCGGATAATATTACCCCTAGTACAATAGTACTAATAAGACTAGTATCCGAA 2520

Hol-I AACCCTGTCTCGGATAATATTACCCCTAGTACAATAGTACTAATAAGACTAGTATCCGAA 2520

************************************************************

Wien-I TAGTATATTTCCCTTGTAGCAAATAATATAATCATAAATGTTCCATATACCAATGGATTT 2580

Hol-I TAGTATATTTCCCTTGTAGCAAATAATATAATCATAAATGTTCCATATACCAATGGATTT 2580

************************************************************

Wien-I AATACCCAACCAATATTAAATAACCTTAAAGTCGTAGGATATTGATAGACCATGCTTTTA 2640

Hol-I AATACCCAACCAATATTAAATAACCTTAAAGTCGTAGGATATTGATAGACCATGCTTTTA 2640

************************************************************

Wien-I TATGTACAAAGTTTACTACACAACCTTAATGCAGTGAGGGACATAATTAATCCTTGTACG 2700

Hol-I TATGTACAAAGTTTACTACACAACCTTAATGCAGTGAGGGACATAATTAATCCTTGTACG 2700

************************************************************

Wien-I GTTCGTACCTACTTGACTCCTCAGTTTAAGCTGAATTGTAGTTTATCGGGACTAAAGTCA 2760

Hol-I GTTCGTACCTACTTGACTCCTCAGTTTAAGCTGAATTGTAGTTTATCGGGACTAAAGTCA 2760

************************************************************

Wien-I GCATAGTCAATTAAAAGGTTTGTTCTCGTAACCATGCTAACACAAATGACATATATATAT 2820

Hol-I GCATAGTCAATTAAAAGGTTTGTTCTCGTAACCATGCTAACACAAATGACATATATATAT 2820

************************************************************

Wien-I ACATTCCGCATTAATTGTGGTTATATAAATCTGTTAATGCTTGTCAAGTTCCTTTTATAT 2880

Hol-I ACATTCCGCATTAATTGTGGTTATATAAATCTGTTAATGCTTGTCAAGTTCCTTTTATAT 2880

************************************************************

Wien-I AGCTATCTCACAGCGTACTTAGGTTCCAACTAAACGAGTTCTCTGTTGGTGTTATATCAC 2940

Hol-I AGCTATCTCACAGCGTACTTAGGTTCCAACTAAACGAGTTCTCTGTTGGTGTTATATCAC 2940

************************************************************

Wien-I AGACTCTGTCTACATATGCTACCCGAACGGTATTCGGTGGTCAATCTATGATCTTAGCTA 3000

Hol-I AGACTCTGTCTACATATGCTACCCGAACGGTATTCGGTGGTCAATCTATGATCTTAGCTA 3000

************************************************************

Wien-I TGGGTTGTATATCTATTCTAGGTGGATTGGTTTGGGCACATCATATGATGACAGTAGGAT 3060

Hol-I TGGGTTGTATATCTATTCTAGGTGGATTGGTTTGGGCACATCATATGATGACAGTAGGAT 3060

************************************************************

Wien-I TAGAAGTAGATACTCGTGCATATTTCTCTGCTGTAACGATTATGATTGCAATACCTACTG 3120

Hol-I TAGAAGTAGATACTCGTGCATATTTCTCTGCTGTAACGATTATGATTGCAATACCTACTG 3120

************************************************************

Wien-I GTACTAAAATATTCAATTGGCTTGGTACTTATATGGCAAGTAACTATGCTACTAGGAATG 3180

Hol-I GTACTAAAATATTCAATTGGCTTGGTACTTATATGGCAAGTAACTATGCTACTAGGAATG 3180

************************************************************

Wien-I TAGATTTATGGGCGGCTCTTAGCTTTATTCTGTTATTTACCTTAGGAGGTACTACAGGTG 3240

Hol-I TAGATTTATGGGCGGCTCTTAGCTTTATTCTGTTATTTACCTTAGGAGGTACTACAGGTG 3240

************************************************************

Wien-I TAGTTATGGGTAATGCAGCTATGGATATAGCATTACACGATACATATTATATTGTAGCTC 3300

Hol-I TAGTTATGGGTAATGCAGCTATGGATATAGCATTACACGATACATATTATATTGTAGCTC 3300

************************************************************

Wien-I ACTTCCATTTTGTGTTATCTTTAGGTGCTGTATTAGCTACAATTTGTGGTTTTGTATATT 3360

Hol-I ACTTCCATTTTGTGTTATCTTTAGGTGCTGTATTAGCTACAATTTGTGGTTTTGTATATT 3360

************************************************************

Wien-I ATGCTAAAGATATGTTTGGAGATACTTTAAACTTCTTCTTACCTGTGACAGGTGGTTCAC 3420

Hol-I ATGCTAAAGATATGTTTGGAGATACTTTAAACTTCTTCTTACCTGTGACAGGTGGTTCAC 3420

************************************************************

Wien-I ATTTCCTAAGTATTTGGTTCTTTGTATTCCTATGTAGTATTATGCTTATCTTCATACCTA 3480

Hol-I ATTTCCTAAGTATTTGGTTCTTTGTATTCCTATGTAGTATTATGCTTATCTTCATACCTA 3480

************************************************************

Wien-I TGCATATATTAGGTTTTAATGTTATGCCTCGAAGGATACCAGATTATCCCGATTTTATTA 3540

Hol-I TGCATATATTAGGTTTTAATGTTATGCCTCGAAGGATACCAGATTATCCCGATTTTATTA 3540

************************************************************

Wien-I CTTATATTAACACAATGTGTTCGATTGGTTCAATCTCTACTATCCTAATCATCTTAGCTA 3600

Hol-I CTTATATTAACACAATGTGTTCGATTGGTTCAATCTCTACTATCCTAATCATCTTAGCTA 3600

************************************************************

Wien-I TGCTTTTATAATACAATAAGATTACAGGCGCATGTAAACTAGTCTTAAACACACCGCTCG 3660

Hol-I TGCTTTTATAATACAATAAGATTACAGGCGCATGTAAACTAGTCTTAAACACACCGCTCG 3660

************************************************************

Wien-I TCACGTAAAAGAATCAATGGTGAGTCCTTTTGGTTGCTCCTTCTGGCTAGACTTTGACTT 3720

Hol-I TCACGTAAAAGAATCAATGGTGAGTCCTTTTGGTTGCTCCTTCTGGCTAGACTTTGACTT 3720

************************************************************

Wien-I GTTCAACCAGCCTGGGATCACAAAATATCTTTGTATGGTAAGATTGAGCGTGGACTATCG 3780

Hol-I GTTCAACCAGCCTGGGATCACAAAATATCTTTGTATGGTAAGATTGAGCGTGGACTATCG 3780

************************************************************

Wien-I AATGAAACAATGTGCTCCACCGCTAGTCTAAGTCTCTAACATACCTTTTACCTTCAACTG 3840

Hol-I AATGAAACAATGTGCTCCACCGCTAGTCTAAGTCTCTAACATACCTTTTACCTTCAACTG 3840

************************************************************

Wien-I TACGGAACGTAACAAACCTCCAGGCAAAGAATAATATTTTGTTCAGCCACTGGTTCACCG 3900

Hol-I TACGGAACGTAACAAACCTCCAGGCAAAGAATAATATTTTGTTCAGCCACTGGTTCACCG 3900

************************************************************

Wien-I TCAACTACCTTGTTTCGACTTCGTACCGACTGTGTTATTGTAGCACATACTCTACCCTAT 3960

Hol-I TCAACTACCTTGTTTCGACTTCGTACCGACTGTGTTATTGTAGCACATACTCTACCCTAT 3960

************************************************************

Wien-I GAAAGGGAGTCTATTCCCAAACAACCGGATCGTGTTGGCTAGGTGAACTAATCACGTTTC 4020

Hol-I GAAAGGGAGTCTATTCCCAAACAACCGGATCGTGTTGGCTAGGTGAACTAATCACGTTTC 4020

************************************************************

Wien-I ATAAATACAATCAGTGAAAGCTCTTTTGATTTCCATGAAGTGAGTTACATATTAGATTCT 4080

Hol-I ATAAATACAATCAGTGAAAGCTCTTTTGATTTCCATGAAGTGAGTTACATATTAGATTCT 4080

************************************************************

Wien-I TTTCGCTCCCTAAGAGTACCGCACCTATGAATATATGACACTCGTACGAGCACATCTAGT 4140

Hol-I TTTCGCTCCCTAAGAGTACCGCACCTATGAATATATGACACTCGTACGAGCACATCTAGT 4140

************************************************************

Wien-I CTATTACAGATGTGCTTTAAATATCAATTTTTCATATAACTTTGGTTTCTTACTTGCTAT 4200

Hol-I CTATTACAGATGTGCTTTAAATATCAATTTTTCATATAACTTTGGTTTCTTACTTGCTAT 4200

************************************************************

Wien-I TGCCTTTGTAATGCAAATTATTACTGGTATTACTTTAGCCCTTCGATATACCTCGGAAGC 4260

Hol-I TGCCTTTGTAATGCAAATTATTACTGGTATTACTTTAGCCCTTCGATATACCTCGGAAGC 4260

************************************************************

Wien-I TTCATACGCCTTTGTTAGCGTACAACATATAGTTAGGGAAGTAGGTGCTGGATGGGAATT 4320

Hol-I TTCATACGCCTTTGTTAGCGTACAACATATAGTTAGGGAAGTAGGTGCTGGATGGGAATT 4320

************************************************************

Wien-I TAGGATGTTACATGCTACTACTGCCTCATGTGTATTCGCGTTTATTTTAGTTCATATGAT 4380

Hol-I TAGGATGTTACATGCTACTACTGCCTCATGTGTATTCGCGTTTATTTTAGTTCATATGAT 4380

************************************************************

Wien-I TCGAGGAATGTACAACAGTAGTTACAGCTATTTAACTACTGCCTGGTTGTCTGGCTTGGT 4440

Hol-I TCGAGGAATGTACAACAGTAGTTACAGCTATTTAACTACTGCCTGGTTGTCTGGCTTGGT 4440

************************************************************

Wien-I TTTATATATTATTACTATTGCAACTGCCTTTCTTGGATATGTGTTACCATGGGGACAAAT 4500

Hol-I TTTATATATTATTACTATTGCAACTGCCTTTCTTGGATATGTGTTACCATGGGGACAAAT 4500

************************************************************

Wien-I GAGTTTTTGGGGAGCCACAGTCATCACCAACCTCTTGTCTCCAATCCCTTACTTAGTACC 4560

Hol-I GAGTTTTTGGGGAGCCACAGTCATCACCAACCTCTTGTCTCCAATCCCTTACTTAGTACC 4560

************************************************************

Wien-I TTGGTTACTAGGAGGGTTTTATGTCTCTGATGTTACCTTAAAACGTTTCTTCGTTTTACA 4620

Hol-I TTGGTTACTAGGAGGGTTTTATGTCTCTGATGTTACCTTAAAACGTTTCTTCGTTTTACA 4620

************************************************************

Wien-I TTTTGTACTTCCTTTTGTAGGTTGTGTTCTAATAGTATTACATATCTTCTATTTACATCT 4680

Hol-I TTTTGTACTTCCTTTTGTAGGTTGTGTTCTAATAGTATTACATATCTTCTATTTACATCT 4680

************************************************************

Wien-I AAACGGTTCTAGTATCCCTGCGG 4703

Hol-I AAACGGTTCTAGTAATCCTGCGG 4703

**************: *******
