# Supplementary figures and images for: Deciphering the code of resistance: a genomic and transcriptomic exploration of the Cystoisospora suis Holland-I strain
Source: Sci Rep. 2025 Feb 14;15:5461. doi: 10.1038/s41598-025-89372-8 (PMC11828913; doi:10.1038/s41598-025-89372-8)

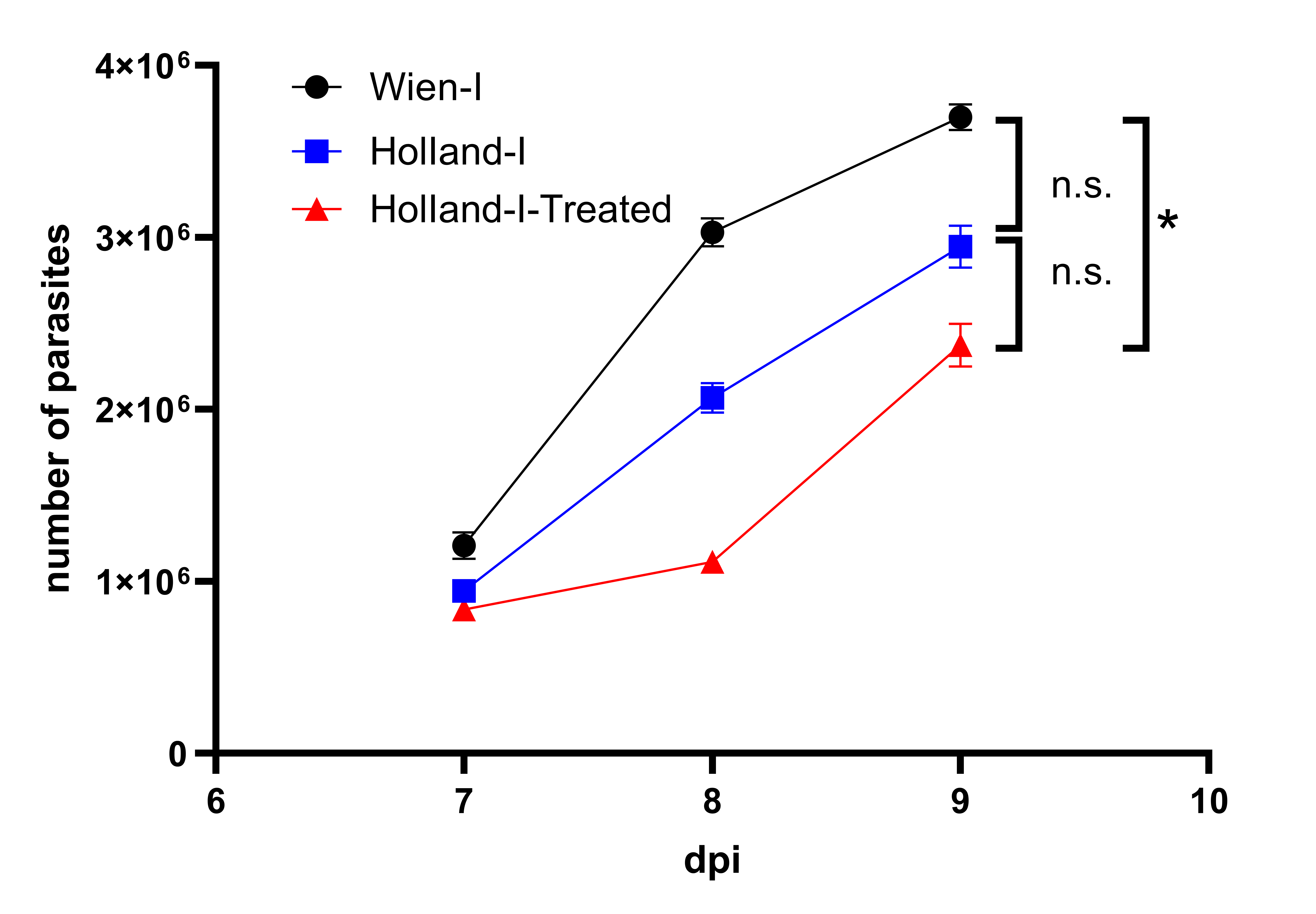

Supplement: Supplementary file 6 — Supplementary Material 6 [file 41598_2025_89372_MOESM6_ESM.tif]
